# Anandins A and B, Two Rare Steroidal Alkaloids from a Marine *Streptomyces anandii* H41-59

**DOI:** 10.3390/md15110355

**Published:** 2017-11-10

**Authors:** Yang-Mei Zhang, Bai-Lian Liu, Xin-Heng Zheng, Xiao-Jun Huang, Hong-Yu Li, Ying Zhang, Ting-Ting Zhang, Da-Yuan Sun, Bi-Run Lin, Guang-Xiong Zhou

**Affiliations:** 1 Guangdong Province Key Laboratory of Pharmacodynamic Constituents of Traditional Chinese Medicine and New Drugs Research, Institute of Traditional Chinese Medicine and Natural Product, College of Pharmacy, Jinan University, Guangzhou 510632, China; yangmei.zhang@chinajijia.com (Y.-M.Z.); tliubl@jnu.edu.cn (B.-L.L.); zhengxinhengxian@sina.com (X.-H.Z.); zhyxiaohuang@jnu.edu.cn (X.-J.H.); hongyu8@ualberta.ca (H.-Y.L.); zhangying@jnu.edu.cn (Y.Z.); tzhtt008@jnu.edu.cn (T.-T.Z.); 2 Key Laboratory of New Technique for Plant Protection in Guangdong, Institute of Plant Protection, Guangdong Academy of Agricultural Sciences, Guangzhou 510632, China; sundayuan@gdaas.cn (D.-Y.S.); yangqy@gdppri.com (B.-R.L.)

**Keywords:** *Streptomyces anandii*, anandins A and B, steroidal alkaloids, cytotoxicity

## Abstract

Anandins A (**1**) and B (**2**), two rare steroidal alkaloids, were isolated from the fermentative broth of a marine actinobacteria *Streptomyces anandii* H41-59. The gross structures of the two alkaloids were elucidated by spectroscopic methods including HR-ESI-MS, and NMR. Their absolute configurations were confirmed by single-crystal X-ray diffraction analysis and comparison of their experimental and calculated electronic circular dichroism spectra, respectively. Anandin A exhibited a moderate inhibitory effect against three human cancer cell lines MCF-7, SF-268, and NCI-H460 with IC_50_ values of 7.5, 7.9, 7.8 μg/mL, respectively.

## 1. Introduction

Because of the increasing difficulty to discover new bioactive compounds from terrestrial sources and the structure diversity of marine metabolites, many researchers have great interest in investigating secondary metabolites from marine-derived organisms [1]. Marine organisms are regarded as a prolific resource of novel bioactive metabolites, including a vast array of macrolide, cyclic peptides, pigments, polyketides, terpenes, steroids and alkaloids, but only a few steroidal alkaloids [2]. Steroidal alkaloids are a class of alkaloids with the basic steroidal skeleton containing a nitrogen atom, either in a ring or in a side chain. Structurally, these alkaloids can be classified into three major groups according to their carbon skeleton, namely, pregnane alkaloids, cholestane alkaloids and C-nor-D-homosteroidal alkaloids. Accumulated evidence in previous studieshas demonstrated that steroidal alkaloids and their glycosides have a wide range of bioactivities, such as antimicrobial, cytotoxic, anti-HIV, anti-inflammation, and antinociceptive activities [3,4,5].

Steroidal alkaloids are well-known metabolites of certain terrestrial plants, mainly from *Solanaceae*, *Liliaceae*, *Apocynaceae* and *Buxaceae* families [6,7,8]. The first steroidal alkaloid was isolated from the berries of *Solanum nigrum* almost 200 years ago [9]. However, steroidal alkaloid was not found from marine organisms until 1984 when two antimicrobial plakinamines were isolated from sponge *Plakina* sp. [10]. Later, four novel cortistatins exhibiting highly selective anti-proliferative activity were isolated from *Corticium* sp. [11], and cytotoxic cephalostatins were discovered from *Cephalodiscus gilchristi* [12]. Compared to those from plants, the marine steroidal alkaloids were relatively few, but with diverse chemistry structures.

*Streptomyces* are known for their ability to produce novel structural and bioactive metabolites [13,14,15]. While searching for bioactive compounds from marine actinomycete, we encountered a strain of actinomycete *Streptomyces anandii* H41-59, the crude extract of which showed strong antifungal activity against *Candida albicans*. In aprevious report, we have isolated three new ergosterols and ten known ones from culture broth of the strain H41-59 [16]. Further investigation on the broth of the same marine actinomycete led to the isolation of two novel ergostane-type steroidal alkaloids, named anandins A (**1**) and B (**2**). In addition, the antibioticand cytotoxic activities of the two steroidal alkaloids were tested in the present study. Herein, details of the isolation, structure elucidation, configuration assignment, and bioactivities of the new metabolites are described.

## 2. Results and Discussion

### 2.1. Elucidation of New Compounds

**Figure 1:**
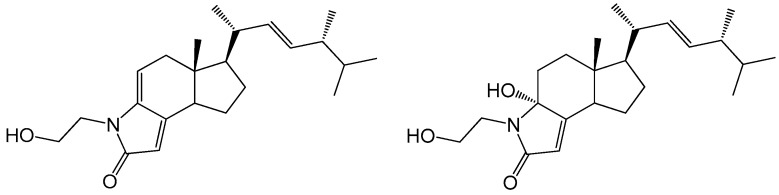
Chemical structure of anandins A (**1**) and B (**2**).

Actinomycete *Streptomyces anandii* H41-59 was isolated from a sea sediment sample from the mangrove zone in the South China Sea. An ethyl acetate partition of the ethanol extract was subjected to silica gel and LH-20 column chromatography, followed by RP-HPLC purification to yield two rare steroidal alkaloids (Figure 1).

Compound **1** was obtained as a colorless plate crystal and given a molecular formula of C_23_H_35_NO_2_. Its seven degrees of unsaturation were determined on the basis of HR-ESI-MS [M + H]^+^ ion at *m*/*z* 358.2752 (C_23_H_36_NO_2_, calcd. 358.2741) (see Figure S1-8). The ^1^H NMR spectrum (see Figure S1) of **1** measured in acetone-*d*_6_ (Table 1) revealed the presence of five characteristic steroid methyl groups including a methyl singlet at *δ*_H_ 0.71 (Me-13) and four methyl doublets at *δ*_H_ 1.08 (Me-15), 0.84 (Me-20), 0.87 (Me-21) and 0.95 (Me-22). The ^13^C NMR and DEPT spectra indicated the presence of 23 carbon signals including a carbonyl carbon at *δ*_C_ 171.1 (C-2), six olefinic carbons at *δ*_C_ 115.3 (C-3), 152.0 (C-4), 141.5 (C-5), 107.4 (C-6), 136.4 (C-16), and 133.1 (C-17), a quaternary carbon at *δ*_C_ 47.0 (C-8), five methine carbons at *δ*_C_ 49.4 (C-9), 55.6 (C-12), 41.1 (C-14), 43.8 (C-18) and 33.9 (C-19), one oxygenated methylene at *δ*_C_ 61.6 (C-2′), four methylenes at *δ*_C_ 40.8 (C-7), 29.7 (C-10), 23.1 (C-11), 20.8 (C-1′), and five methyl carbons at *δ*_C_ 12.4 (C-13), 21.5 (C-15), 20.1 (C-20), 20.4 (C-21) and 18.2 (C-22). Further analyses of the 1D NMR and HSQC data suggested that compound **1** was more likely to be a highly degraded sterol.

**Figure 2:**
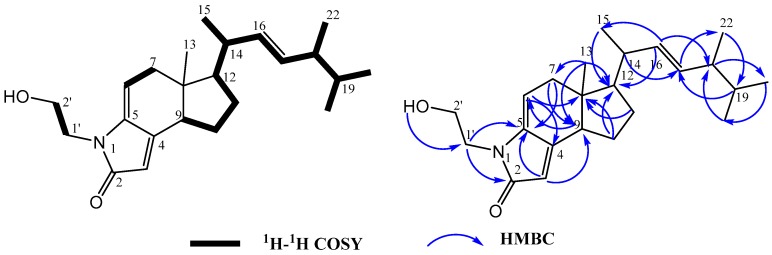
Key ^1^H-^1^H COSY and HMBC correlations of **1**.

The H-H COSY spectrum of **1** gave several spin systems which belong to substructures as shown in Figure 2 with bold lines. The HMBC correlations from a sp^2^methine proton at *δ*_H_ 5.27 (1H, dd, H-16) to carbons at *δ*_C_ 21.5 (C-15, ^3^*J*) and 43.8 (C-18, ^3^*J*), and from a sp^2^methine proton at *δ*_H_ 5.30 (1H, dd, H-17) to carbon at *δ*_C_ 41.1 (C-14, ^3^*J*), confirmed the presence of a characteristic side chain of ergosterol [16]. A methyl proton at *δ*_H_ 0.71 (3H, s, Me-13) displayed four HMBC correlations to carbons at *δ*_C_ 40.8 (C-7, ^3^*J*), 47.7 (C-8, ^2^*J*), 49.4 (C-9, ^3^*J*) and 55.6 (C-12, ^3^*J*), interpreted as direct attachment to the quaternary C-8. The HMBC correlations from the proton at *δ*_H_ 1.93 (1H, m, H-10) to carbons at *δ*_C_ 47.7 (C-8, ^3^*J*) and 55.6 (C-12, ^3^*J*), from the proton at *δ*_H_ 5.60 (1H, m, H-6) to carbons at *δ*_C_ 47.7 (C-8, ^3^*J*) and 152.0 (C-4, ^3^*J*), from protons at *δ*_H_ 2.63 (2H, m, H-7)/2.34 (dd, 2.5, 17.5) to carbons at *δ*_C_ 141.5 (C-5, ^3^*J*), 49.4 (C-9, ^3^*J*) and 55.6 (C-12, ^3^*J*), confirmed the substructure of the B ring and C ring of ergosterol. The HMBC correlations from the proton at *δ*_H_ 5.63 (1H, br.s, H-3) to carbons at *δ*_C_ 171.1 (C-2, ^2^*J*), 141.5 (C-5, ^3^*J*) and 49.4 (C-9, ^3^*J*), from protons at *δ*_H_ 3.64 (2H, m, H-1′) to carbons at 171.1 (C-2, ^3^*J*) and 141.5 (C-5, ^3^*J*), and from protons at *δ*_H_ 3.63 (2H, m, H-2′) to carbon at *δ*_H_ 171.1 (C-2, ^4^*J*), confirmed the presence of an α,β-unsaturated γ-lactam and two methylene carbons linked to γ-lactam and the hydroxyl group.

**Figure 3:**
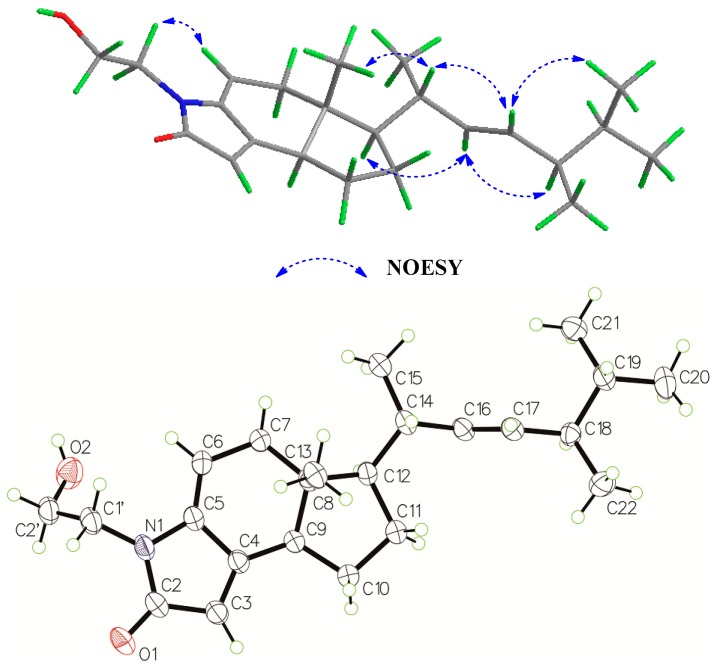
Key NOESY correlations and X-ray ORTEP of **1**.

The relative configuration of **1** was determined on the basis of the analysis of ^1^H-^1^H coupling constants and the NOESY information (Figure 3). Large coupling constants of 15.1 Hz between H-16 and H-17, and the NOESY correlations between H-17 and H-14, between H-17 and H-20/H-21, between H-16 and H-12, and between H-16 and H-18 indicated the *E* configuration of the double bond in the side chain. A correlation was also observed between H-13β and H-14, confirming the β-orientation of H-14. The absolute configuration of compound **1** was unequivocally defined as (16*E*,8*R*,12*R*,14*S*,18*R*) by single crystal X-ray diffraction analysis (see Table S1) using Cu K*a* radiation with Flack and Hooft parameters of 0.1 (2) and 0.15 (6), respectively. Compound **1** is a new steroidal alkaloid, named as anandin A. 

**Figure 4:**
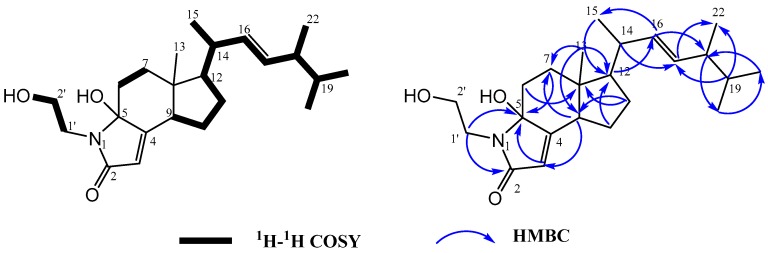
Key 2D NMR correlations of **2**.

Compound **2** was isolated as white powder. Its molecular formula was determined as C_23_H_37_NO_3_ with six degrees of unsaturation, by the HR-ESI-MS [M + H]^+^ ion at *m*/*z* 376.2860 (C_23_H_38_NO_3_, calcd. 376.2853) (see Figure S2-8). The ^13^C NMR and DEPT (see Figure S2) spectroscopic data of **2** measured in acetone-*d*_6_ (Table 1) indicated the presence of 23 carbon signals for a carbonyl carbon at *δ*_C_ 171.1 (C-2), four olefinic carbons at *δ*_C_ 116.5 (C-3), 164.9 (C-4), 136.4 (C-16), 133.1 (C-17), one quaternary signal at *δ*_C_ 49.1 (C-8), one oxygenated methylene at *δ*_C_ 62.3 (C-2′), five methine carbons at *δ*_C_ 50.1 (C-9), 56.3 (C-12), 41.2 (C-14), 43.8 (C-18) and 33.9 (C-19), five methyl groups at *δ*_C_ 12.1 (C-13), 21.5 (C-15), 20.1 (C-20), 20.4 (C-21) and 18.2 (C-22). The ^1^H and ^13^C NMR spectroscopic data of **2** were comparable to those of **1**, suggesting that **2** is an analogue of **1** with an additional oxygenated quaternary carbon and the absence of a double bond. The gross structure of **2** was further elucidated by analysis of COSY and HMBC spectrum data (Figure 4). The substructure of γ-hydroxy-α,β-unsaturated γ-lactam was deduced by the HMBC correlations from H-3 (*δ*_H_ 5.52, br.s, 1H) to C-2 (*δ*_C_ 171.1, ^2^*J*) and C-5 (*δ*_C_ 89.3, ^3^*J*), from H-1′ (*δ*_H_ 3.60, 3.32) to C-2 (*δ*_C_ 171.1, ^3^*J*) and C-5 (*δ*_C_ 89.3, ^3^*J*), from H-2′ (*δ*_H_ 3.66, m, 2H) to C-2 (*δ*_C_ 171.1, ^4^*J*), and from H-7 (*δ*_H_ 2.23/1.60, m, 2H) to C-5 (*δ*_C_ 89.3, ^3^*J*). The HMBC correlation from H-9 (*δ*_H_ 2.65, m, 1H) to C-5 (*δ*_C_ 89.3, ^3^*J*), C-3 (*δ*_C_ 116.5, ^3^*J*) and C-12 (*δ*_C_ 56.3, ^3^*J*), from H-11 (*δ*_H_ 1.65/1.58, m, 2H) to C-9 (*δ*_C_ 50.1, ^3^*J*), from H-9 (*δ*_H_ 2.65, m, 1H) to C-5 (*δ*_C_ 89.3, ^3^*J*) and C-3 (*δ*_C_ 116.5, ^3^*J*), confirmed the substructure of the B ring and the saturated C ring of ergosterol. Other HMBC correlations permitted further confirmation of the carbon skeleton of **2**.

**Figure 5:**
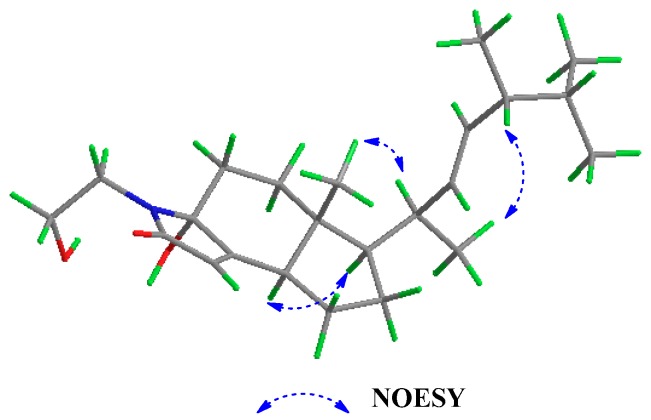
Key NOESY correlations of **2**.

**Figure 6:**
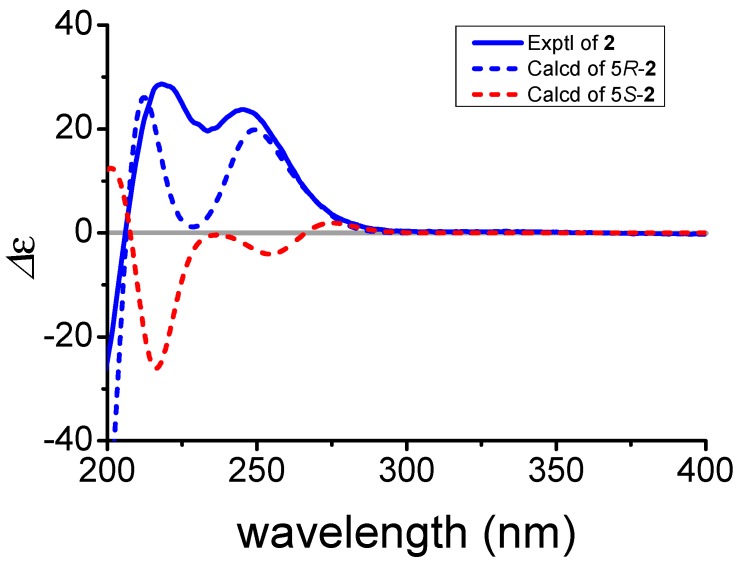
Experimental and calculated ECD spectra of **2**.

In the NOESY spectrum, NOE correlations (Figure 5) of H-13 with H-14, and H-9 with H-12, confirmed the *β*-orientation of the side chain and the *α*-orientation of H-12, respectively. A large coupling constant (14.7 Hz) between H-16 and H-17 indicated the *E* configuration of the double bond between C-16 and C-17. The relative configuration at C-14 and C-18 was tentatively assigned to be identical to those in **1** based on similar NMR spectrum data and biogenetic consideration. The orientation of 5-OH (*δ*_H_ 5.12) was tentatively defined to be *α*, because the comparison of the experimental and calculated CD spectra (Figure 6) facilitated assignment of the absolute configuration of **2** as 16*E*,5*R*,8*R*,12*R*,14*S*,18*R*. Compound **2** is named as anandin B.

Anandins A (**1**) and B (**2**) are two rare steroidal alkaloids possessing a unique highly degraded ergosterol skeleton and a conjugated γ-lactam moiety. Why do two novel conjunction-type steroidal alkaloids exist in the ethanol extract of the mycelium material of *Streptomyces anandii* H41-59? With further investigation, we found that a cholesterol oxidase (Cho), usually observed among Gram-positive G+C-rich actinobacteria, is able to oxidize and degrade steroids [17]. In a previous study, we discovered a series of ergosterols including three new ones [16]. Thus, we considered that anandins A (**1**) and B (**2**) were probably derived from a highly degraded ergosterol [18,19]. 

### 2.2. Bioactivities of ***1*** and ***2***

Anandins A (**1**) and B (**2**) were evaluated in a wide panel of biological assays, including antimicrobial activity against *Candida albicans*, *Escherichia coli*, *Staphylococcus aureus*, *Bacillus* sp. and *Dickey azeae*, and anti-proliferative activity against human breast adenocarcinoma cell line MCF-7, human glioblastoma cell line SF-268 and human lung cancer cell line NCI-H460 by the methods described below. As shown in Table 2, both compounds **1** and **2** were shown to be active against three cancer cell lines. As a result, compound **1** exhibited moderate cytotoxicity against MCF-7, SF-268 and NCI-H460 with IC_50_ values of 7.5, 7.9, 7.8 μg/mL, respectively. However, both the compounds were inactive against the tested strains at the concentration of 20 μg/mL, even if the crude extract displayed moderate antimicrobial activity (inhibiting the zone of 16 mm against *C. albicans* with 6 mm paper discs at the concentration of 20 μg/mL).

## 3. Experimental Section

### 3.1. General

Melting points were measured on an X-5 micro-MP apparatus (Huayan Corporation, Shanghai, China), uncorrected. Optical rotations were measured with a JASCO digital polarimeter (JASCO Corporation, Tokyo, Japan). UV spectra were measured on a JASCO V-550 UV/VIS spectrometer (JASCO Corporation, Tokyo, Japan). IR data were recorded with a Nicolet Impact 410-FTIR instrument (Thermo, San Jose, CA, USA) in KBr pellets. HR-ESI-MS were acquired on an Agilent 6210 LC/MSD TOF mass spectrometer (Agilent Technologies, Santa Clara, CA, USA). NMR spectra were measured on a Bruker AV-300 and AV-600 spectrometer (Bruker Instrument, Inc., Zurich, Switzerland). Chemical shifts were expressed in *δ* (ppm) and referenced to the NMR solvent used. X-ray crystallographic analysis was performed on an X calibur, sapphires, Gemini ultra diffractometer (Oxford Diffraction Ltd., Tokyo, Japan). The crystal was kept at 173.00(10) K during data collection. Using Olex2, the structure was solved with the ShelXS structure solution program using Direct Methods and refined with the ShelXL refinement package using Least Squares minimisation. HPLC was performed on an Agilent 1200 HPLC system (Agilent Technologies, Palo Alto, CA, USA) equipped with a diode array detector, using a column A (Ultimate XB-C18, 5 μm, 4.6 × 250 mm, Welch, Potamac, MA, USA) for analysis and a semi-preparative HPLC column B (Ultimate XB-C18, 5 μm, 10 × 250 mm, Welch, Potamac, MA, USA) for purification. Open column chromatography was performed on silica gel (300–400 mesh, Qingdao Haiyang Chemical Group Corporation, Qingdao, China). Sephadex LH-20 (25–100 mm) was purchased from Pharmacia (Uppsala, Sweden). HSGF254 silica gel TLC plates (0.2 mm thickness, 200 × 200 mm, Qingdao Marine Chemicals Co., Qingdao, China) were used for routine analysis of fractions. Strains *Candida albicans*, *Escherichia coli*, *Staphylococcus aureus*, *Bacillus* sp., and *Dickey azeae* were from the Institute of New Drug Research (Guangzhou, China) in our college.

### 3.2. Strain Isolation and Identification

The isolation and activation of actinomycete strain H41-59; the identification of morphological characteristics, physiological and chemical properties; the molecular genetic analysis and information on storing the strain are mentioned in our previous report [16].

### 3.3. Fermentation and Isolation

Fermentation of strain H41-59 including condition and media compositions were reported in our earlier paper [16]. The extract and partition procedure from fermentation material for the EtOAc extract were introduced in the same paper. The ethyl acetate-soluble extract (50 g) was dissolved in chloroform and loaded on the silica gel column (1.5 kg, 300–400 mesh, Qingdao, China) after filtration. A stepwise gradient elution of petroleum ether-EtOAc (10:0, 9:1, 8:2, 7:3, 6:4, 5:5, 4:6, 2:8 and 0:10 (*v*/*v*)) was used, and 20 fractions (Fr-1 to Fr-20) were obtained through TLC analysis and combination of fractions with same TLC pattern. Fr-6 was separated on Sephadex LH-20 (2 × 200 cm, CH_2_Cl_2_-MeOH, 1:1) and purified via semi-preparative HPLC (MeOH-H_2_O, 90:10) to yield two new natural products, namely, **1** (10 mg) and **2** (3 mg).

Compound **1** (*anandin A*): colorless plate crystal; M.P. = 133–135 °C; ${\left[\alpha \right]}_{D}^{23}$ −13.5° (*c* 0.25, CHCl_3_); UV (MeOH) *λ*_max_ (log*ε*): 204.6 (3.88), 263.8 (3.73) nm; IR (KBr) ν_max_: 3382, 2958, 2930, 2871, 1666, 1650, 1372, 1300, 1191, 1111, 1015, 981, 620 cm^−1^; ^1^H NMR (CD_3_COCD_3_, 300 MHz) and ^13^C NMR (CD_3_COCD_3_, 75 MHz), see Table 1; HR-TOF-ESI-MS (positive) *m*/*z* 358.2752 [M + H]^+^ (calcd. for C_23_H_36_NO_2_, 358.2741).

Compound **2** (*anandin B*): white powder; M.P. = 129–131 °C; ${\left[\alpha \right]}_{D}^{23}$ −15.1° (*c* 0.25, CHCl_3_); UV (MeOH) λ_max_ (logε): 208.2 (3.78) nm; IR (KBr) ν_max_: 3423, 2929, 2853, 1650, 1542, 1461, 1381, 1190, 1110, 620 cm^−1^; ^1^H NMR (CD_3_COCD_3_, 600 MHz) and ^13^C NMR (CD_3_COCD_3_, 150 MHz), see Table 1; HR-ESI-MS: *m*/*z* 376.2860 [M + H]^+^ (calcd. for C_23_H_38_NO_3_, 376.2846).

### 3.4. Single-Crystal X-ray Data for Anandin A *(**1**)*

Crystal data (CCDC No. 1544792) for **1**: C_23_H_35_NO_2_, *M* = 357.52, *T* = 100(2) K, monoclinic, space group *C*2, *a* = 21.7844 (3) Å, *b* = 7.2393 (11) Å, *c* = 13.1475 (20) Å, *α* = 90.00°, *β* = 93.2128 (13)°, *γ* = 90.00°, *V* = 2070.15 (5) Å^3^, *Z* = 4, *μ* (Cu K*α*) = 0.555 mm^−1^, 16,181 reflections measured, 3274 independent reflections (R_int_ = 0.0282). The final *R*_1_ values were 0.0286 (I > 2σ (*I*)). The final *wR* (*F*^2^) values were 0.0747 (I > 2σ (*I*)). The final *R*_1_ values were 0.0290 (all data). The final *wR* (*F*^2^) values were 0.0750 (all data). The goodness of fit on *F*^2^ was 1.058. Flack parameter = 0.1 (2). Hooft parameter = 0.15 (6).

### 3.5. ECD Calculation

*ECD calculations of Anandium B* (**2**)*:* The systematic random conformational analysis of two possible stereoisomers (**5*R*** and **5*S***) of **2** was performed in the SYBYL 8.1 program by using a MMFF94s molecular force field, which afforded 40 and 42 conformers for **5*R*** and **5*S*** respectively, with an energy cutoff of 10 kcal mol^−1^ to the global minima. All the obtained conformers were further optimized using DFT at the B3LYP/6-31+G (d) level in gas phase by using Gaussian09 software (Gaussian, Inc., Wallingford, CT, USA), and eight conformers of each stereoisomer were selected. All of the optimized stable conformers were used for TDDFT (cam-B3LYP/6-31+G (d)) computation of the excited states at the same levels, with consideration of the first 50 excitations. The overall ECD curves were weighted by the Boltzmann distribution of each conformer (with a half-band width of 0.25 eV), with a UV correction of 10 nm. The calculated ECD spectra of **5*R*** and **5*S*** of **2** were subsequently compared with the experimental spectra, respectively. The ECD spectra were produced by SpecDis 1.6 software (T. Bruhn, Y. Hemberger, A. Schaumlöffel, G. Bringmann, SpecDis version 1.6, University of Wuerzburg, Wuerzburg, Germany).

### 3.6. Biological Activities

#### 3.6.1. Antimicrobial Activity

Antimicrobial evaluation of compounds **1** and **2** was performed following the same method and procedure as in a previous paper [16].

#### 3.6.2. Cytotoxicity Assay

Three cancer cell lines MCF-7, SF-268, and NCI-H460 were used for measuring the cytotoxicities of compounds **1** and **2**. For details on the procedure, we refer readers to our previous paper [16]. The 50% inhibition concentrations (IC_50_) of compounds **1** and **2** against the test cells were calculated using Origin 8 software (OriginLab, Northampton, MA, USA).

## 4. Conclusions

Anandins A (**1**) and B (**2**) were obtained from the fermented mycelia of *Sterptomyces anandii* H41-59. They have the same basic skeleton, and belong to a rare type of steroidal alkaloids. The structures of **1** and **2** were elucidated on the basis of extensive spectroscopic data including HR-ESI-MS, NMR, and X-ray crystallography for **1**, and calculation chemistry for **2**. To the best of our knowledge, **1** and **2** are the second reported compounds and second and third compounds with this type of structure. Anandin A (**1**) exhibited moderate *in vitro* inhibitory activity against MCF-7, SF-268, and NCI-H460 cell lines by the MTT method. Neither **1** nor **2** showed any antibiotic activities against the test pathogenic microorganisms.

## Figures and Tables

**Table 1:** ^1^H and ^13^C NMR data of **1** and **2** (*δ* in ppm, *J* in Hz).

Position	1 ^a^		2 ^b^	
	*δ*_H_	*δ*_C_	*δ*_H_	*δ*_C_
2	-	171.1	-	171.1
3	5.63 (br.s)	115.3	5.52 (d, 1.9)	116.5
4	-	152.0	-	164.9
5	-	141.5	-	89.3
6	5.60 (m)	107.4	1.65 (m), 1.92 (m)	36.0
7	2.63 (m), 2.34 (dd, 2.5, 17.5)	40.8	2.23 (m), 1.60 (m)	35.7
8	-	47.0	-	49.1
9	2.68 (m)	49.4	2.65 (m)	50.1
10	1.48 (m), 1.93 (m)	29.7	1.46 (m), 1.91 (m)	29.6
11	1.51 (m), 1.94 (m)	23.1	1.58 (m), 1.65 (m)	22.0
12	1.59 (m)	55.6	1.53 (m)	56.3
13	0.71 (s)	12.4	0.60 (s)	12.1
14	2.13 (m)	41.1	2.11 (br. s)	41.2
15	1.08 (d, 6.9)	21.5	1.08 (d, 6.9)	21.5
16	5.27 (dd, 7.7, 15.1)	136.4	5.28 (dd, 7.6, 14.6)	136.4
17	5.30 (dd, 7.2, 15.1)	133.1	5.30 (dd, 7.2, 14.6)	133.1
18	1.90 (m)	43.8	1.90 (m)	43.8
19	1.51 (m)	33.9	1.50 (m)	33.9
20	0.84 (d, 6.9)	20.1	0.85 (d, 6.9)	20.1
21	0.87 (d, 6.9)	20.4	0.88 (d, 6.9)	20.4
22	0.95 (d, 6.8)	18.2	0.96 (d, 6.9)	18.2
1′	3.64 (m)	42.8	3.32 (m), 3.60 (m)	42.5
2′	3.63 (m)	61.1	3.66 (m)	62.3
5-OH			5.12 (br.s)	-
2′-OH	3.87 (br.s)	-	4.36 (br.s)	-

^a^ Measured in CD_3_COCD_3_ at 300 MHz for ^1^H and 75 MHz for ^13^C NMR; ^b^ Measured in CD_3_COCD_3_ at 600 MHz for ^1^H and 125 MHz for ^13^C NMR.

**Table 2:** Cytotoxicities of **1** and **2** (IC_50_: μg/mL).

Cell Line	1	2	*cis*-Dichlorodiamine Platinum
MCF-7	7.5	>50	4.0
SF-268	7.9	>50	41.0
NCI-H460	7.8	>50	25.1

Concentration range: 1.56–100 μg/mL; IC_50_: half maximal inhibitory concentration.

## Supplementary Materials

The following are available online at http://www.mdpi.com/1660-3397/15/11/355/s1, Figure S1: Spectra of compound **1**; Figure S2: Spectra of compound **2**; Table S1: X-ray diffraction data compound **1**.

## Abbreviations

COSY	Two dimensional ^1^H correlation
DEPT	Distortionless enhancement by polarization transfer
HPLC	High performance liquid chromatography
HMBC	^1^H-detected heteronuclear multiple-bond correlation
HR-ESI-MS	High resolution electrospray ionization mass spectrometry
HSQC	^1^H-detected heteronuclear single-quantum coherence
MTT	3-(4,5-dimethyl-2-thiazolyl)-2,5-diphenyl-2-H-tetrazolium bromide
NMR	Nuclear magnetic resonance
NOESY	Nuclear overhauser effect spectroscopy
TLC	Thin layer chromatography
