# Supplementary material for: Anandins A and B, Two Rare Steroidal Alkaloids from a Marine Streptomyces anandii H41-59
Source: Mar Drugs. 2017 Nov 10;15(11):355. doi: 10.3390/md15110355 (PMC5706044; doi:10.3390/md15110355)
Supplement: Supplementary file 1 [file marinedrugs-15-00355-s001.pdf]

Supporting material

Anandins A and B, Two Rare Steroidal Alkaloids from a Marine *Streptomyces anandii* H41-59

Yang-Mei Zhang, Bai-Lian Liu, Xiao-Jun Huang, Hong-Yu Li, Ying Zhang, Tingting Zhang, Dayuan Sun, Bi-run Lin, Guang-Xiong Zhou

Abstract: Anandins A (**1**) and B (**2**), two rare steroidal alkaloids, were isolated from the fermentative broth of a marine actinobacteria *Streptomyces anandii* H41-59. The gross structures of the two alkaloids were elucidated by spectroscopic methods including HR-ESI-MS, and NMR. Their absolute configurations were confirmed by single-crystal X-ray diffraction analysis and comparison of their experimental and calculated electronic circular dichroism spectra, respectively. Anandin A exhibited a moderate inhibitory effect against three human cancer cell lines MCF-7, SF-268, and NCI-H460 with IC_{50} value of 7.5, 7.9, 7.8 μg/mL, respectively.

Keywords: *Streptomyces anandii*; Anandins A and B; steroidal alkaloids; cytotoxicity

List：

Figure S1. Spectra of compound **1**

Figure S1-1 ^{1}H NMR spectrum of compound **1** (300 MHz, in CD_3COCD_3) 2

Figure S1-2 ^{13}C NMR spectrum of compound **1** (75 MHz, in CD_3COCD_3) 2

Figure S1-3 DEPT-135 spectrum of compound **1** (75 MHz, in CD_3COCD_3)........ 3

Figure S1-4 ^{1}H-^{1}H COSY spectrum of compound **1** (300 MHz, in CD_3COCD_3) 3

Figure S1-5 HMQC spectrum of compound **1** (300 MHz, in CD_3COCD_3)........ 4

Figure S1-6 HMBC spectrum of compound **1** (300 MHz, in CD_3COCD_3)........ 4

Figure S1-7 NOESY spectrum of compound **1** (300 MHz, in CD_3COCD_3) 5

Figure S1-8 HR-ESI-MS of compound **1** 5

Figure S1-9 X-ray structure of compound **1** 6

Figure S1-10 IR spectrum of compound **1**........ 6

Figure S1-11 UV spectrum of compound **1** 6

Figure S2. Spectra of compound **2**

Figure S2-1 ^{1}H NMR spectrum of compound **2** (600 MHz, in CD_3COCD_3) 7

Figure S2-2 ^{13}C NMR spectrum of compound **2** (150 MHz, in CD_3COCD_3) 7

Figure S2-3 DEPT-135 spectrum of compound **2** (150 MHz, in CD_3COCD_3)........ 8

Figure S2-4 ^{1}H-^{1}H COSY spectrum of compound **2** (600 MHz, in CD_3COCD_3) 8

Figure S2-5 HMQC spectrum of compound **2** (600 MHz, in CD_3COCD_3)........ 9

Figure S2-6 HMBC spectrum of compound **2** (600 MHz, in CD_3COCD_3)........ 9

Figure S2-7-A NOESY spectrum of compound **2** (600 MHz, in CD_3COCD_3) 10

Figure S2-7-B ROESY spectrum of compound **2** (600 MHz, in CD_3COCD_3)........ 10

Figure S2-8 HR-ESI-MS of compound **2** 11

Figure S2-9 IR spectrum of compound **2**........11

Figure S2-10 UV spectrum of compound **2** 11

Table S1. X-ray crystallograpgic data of Compound **1**........ 12

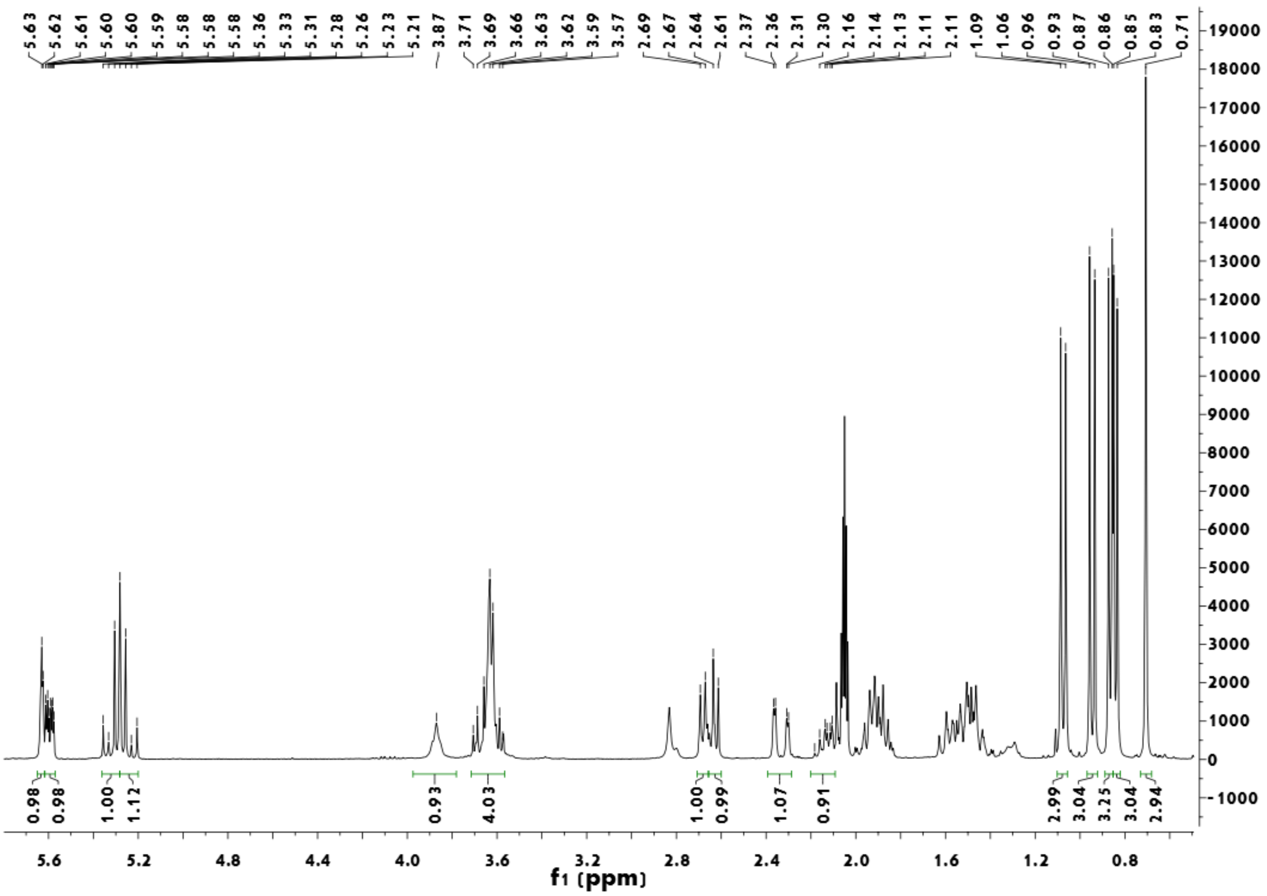

Figure S1-1 ^{1}H NMR spectrum of compound **1** (300 MHz, in CD_3COCD_3)

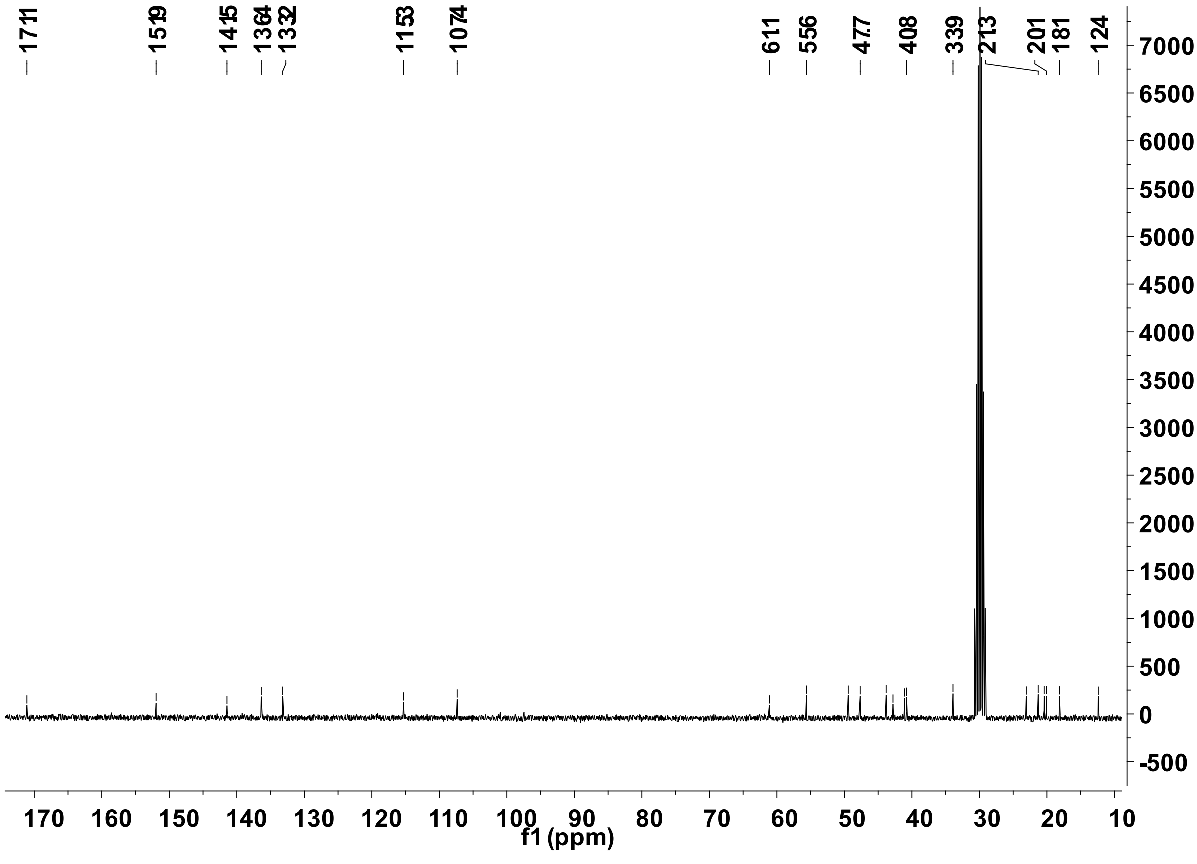

Figure S1-2 ^{13}C NMR spectrum of compound **1** (75 MHz, in CD_3COCD_3)

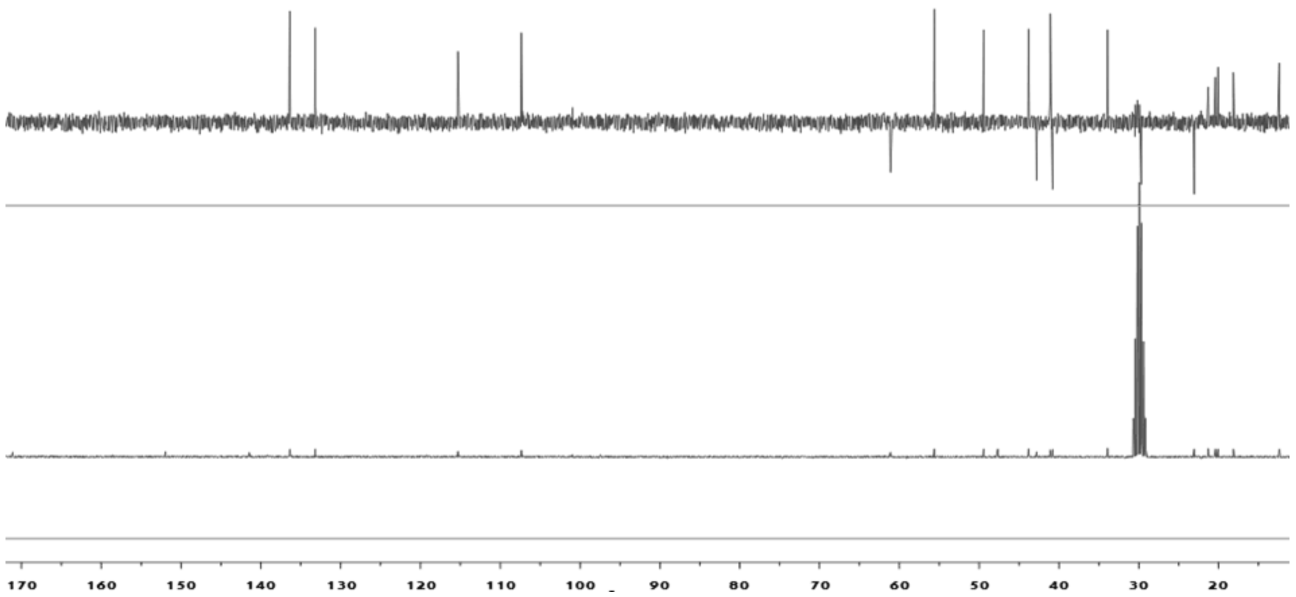

Figure S1-3 DEPT-135 spectrum of compound **1** (75 MHz, in CD_3COCD_3)

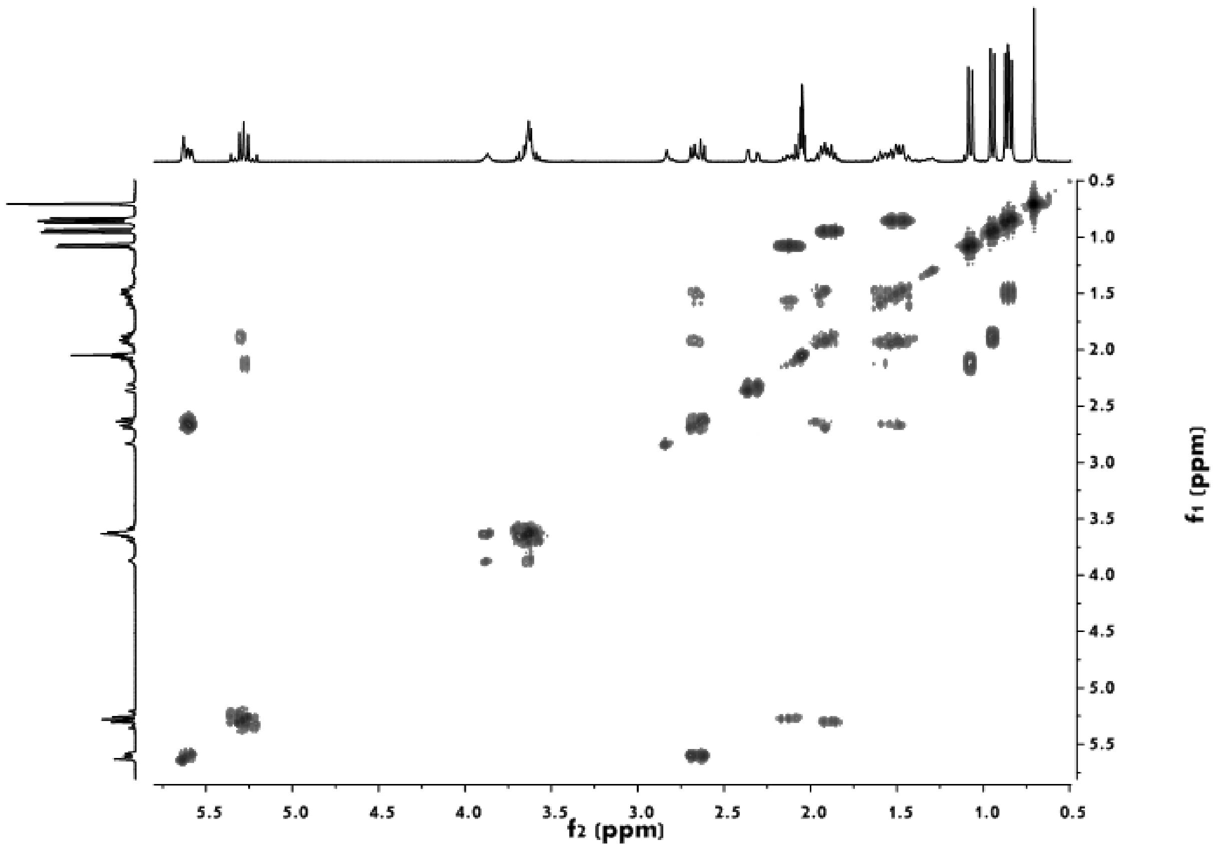

Figure S1-4 ^1H-^1H COSY spectrum of compound **1** (300 MHz, in CD_3COCD_3)

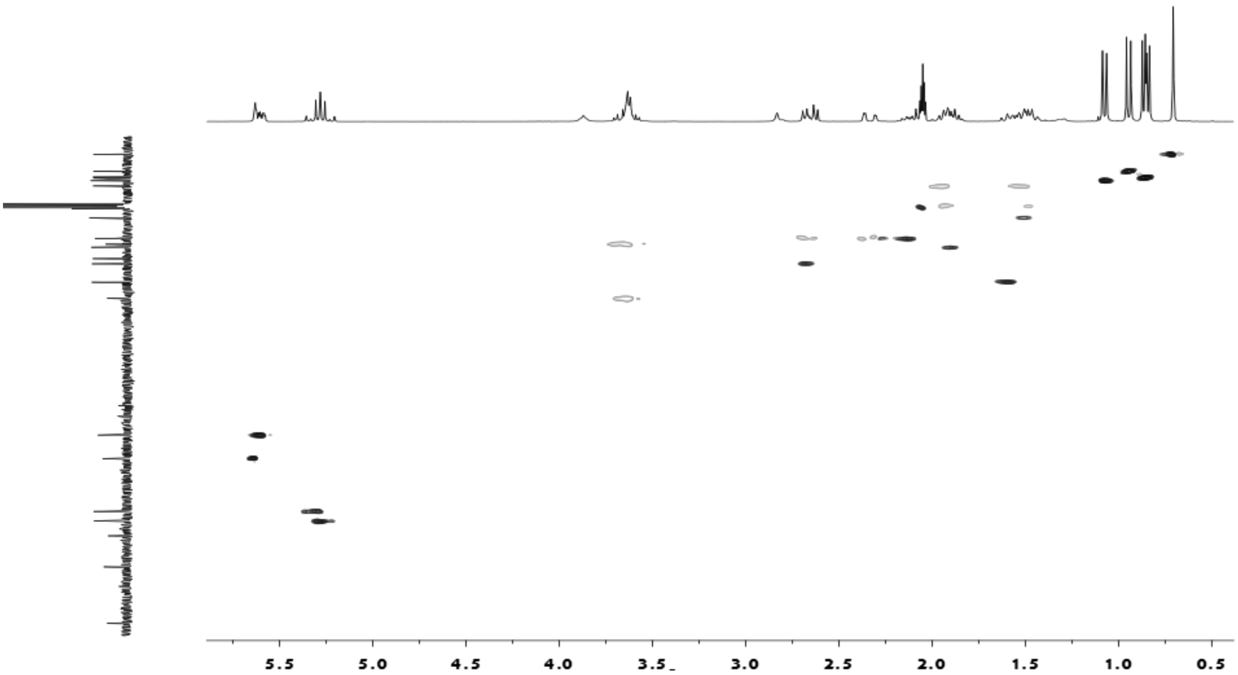

Figure S1-5 HMQC spectrum of compound **1** (300 MHz, in CD_3COCD_3)

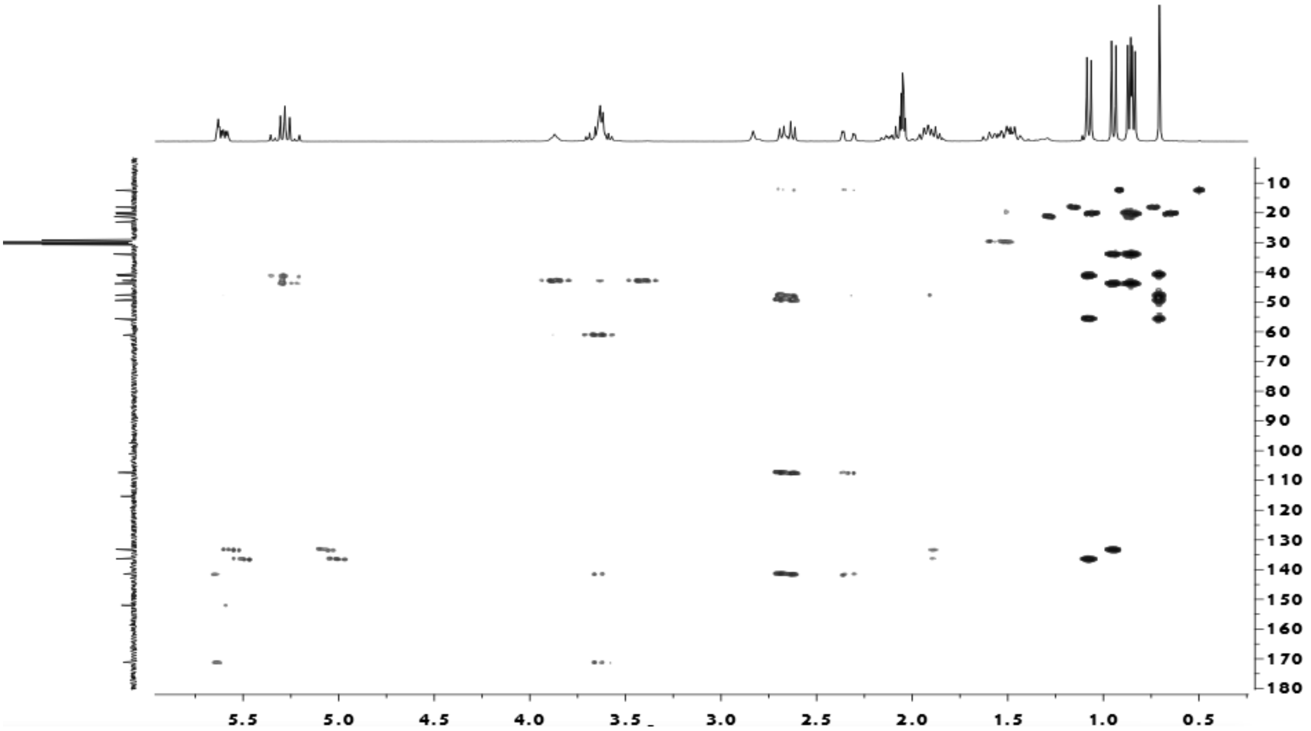

Figure S1-6 HMBC spectrum of compound **1** (300 MHz, in CD_3COCD_3)

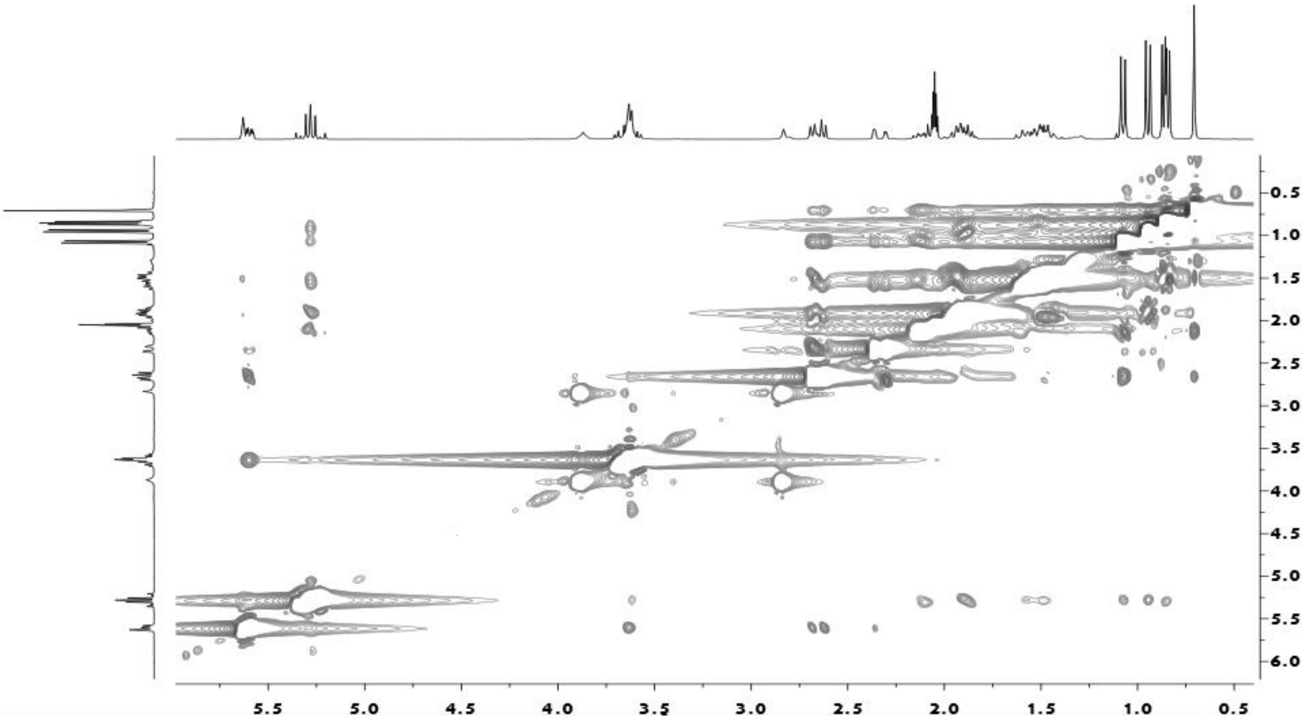

Figure S1-7 NOESY spectrum of compound **1** (300 MHz, in CD_3COCD_3)

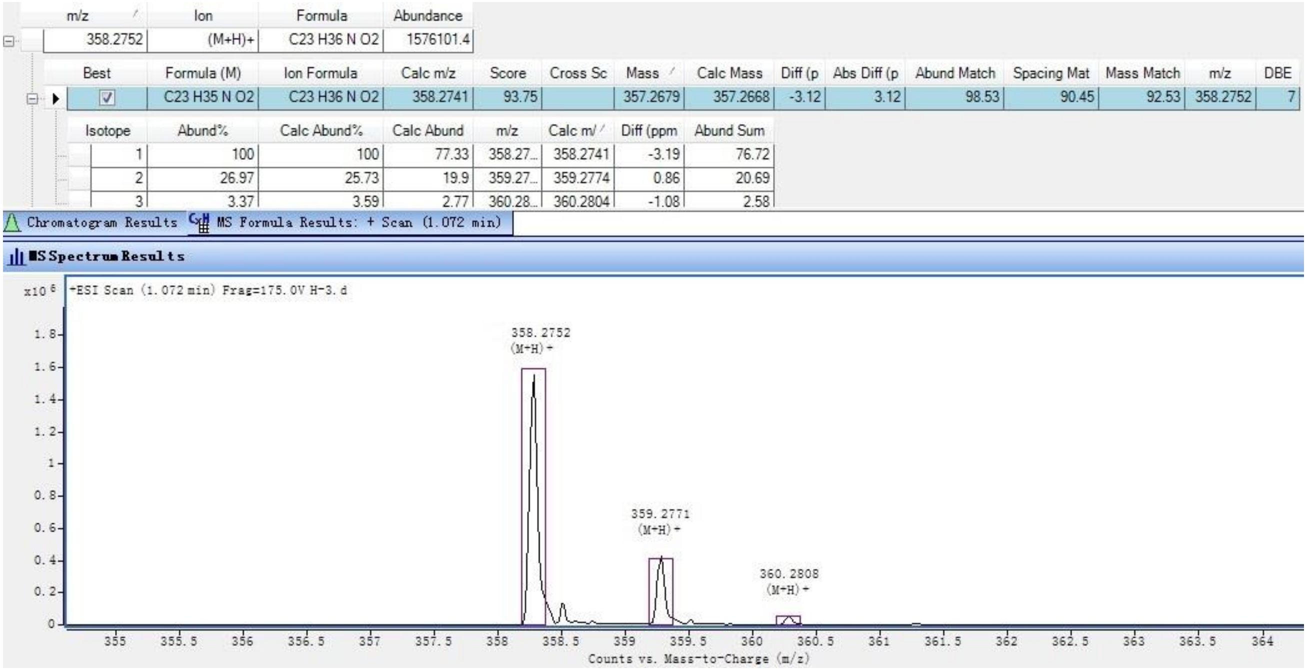

m/z	Ion	Formula	Abundance
358.2752	(M+H)+	C23 H36 N O2	1576101.4

Best	Formula (M)	Ion Formula	Calc m/z	Score	Cross Sc	Mass	Calc Mass	Diff (p	Abs Diff (p	Abund Match	Spacing Mat	Mass Match	m/z	DBE
☑	C23 H35 N O2	C23 H36 N O2	358.2741	93.75		357.2679	357.2668	-3.12	3.12	98.53	90.45	92.53	358.2752	7

Isotope	Abund%	Calc Abund%	Calc Abund	m/z	Calc m/	Diff (ppm	Abund Sum
1	100	100	77.33	358.27...	358.2741	-3.19	76.72
2	26.97	25.73	19.9	359.27...	359.2774	0.86	20.69
3	3.37	3.59	2.77	360.28...	360.2804	-1.08	2.58

Figure S1-8 HR-ESI-MS of compound **1**

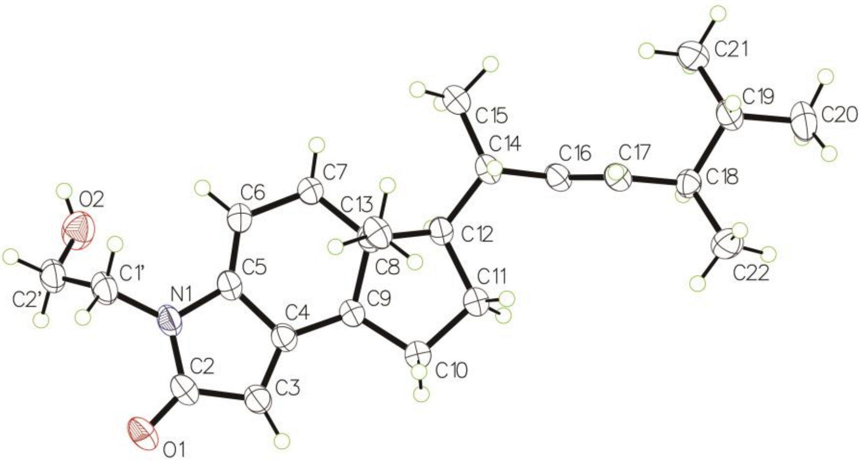

Figure S1-9 X-ray structure of compound **1**

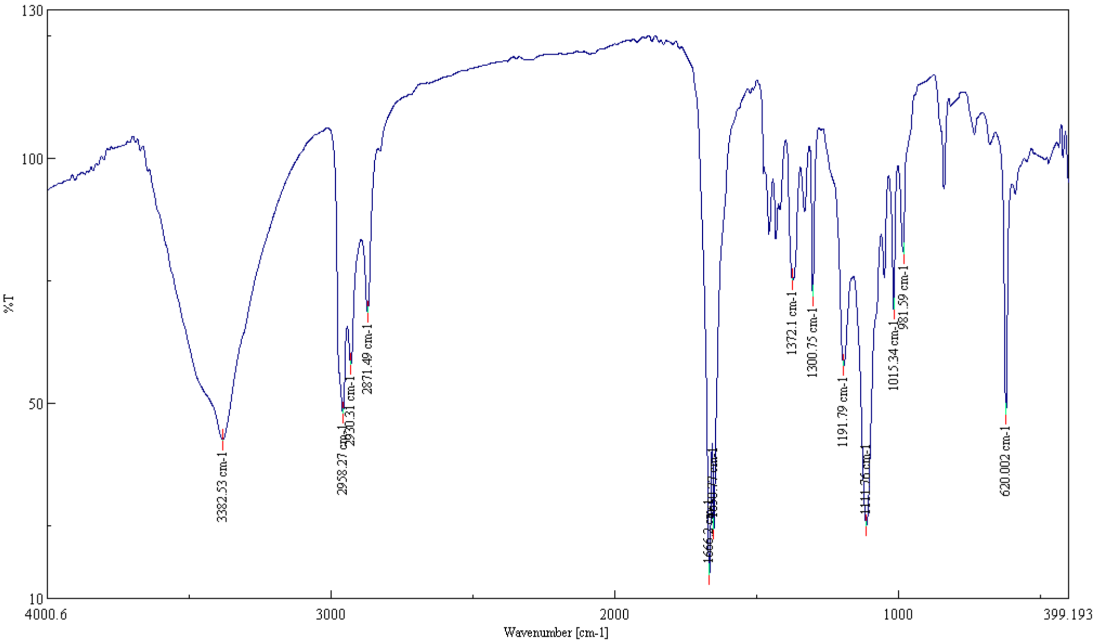

Figure S1-10 IR spectrum of compound **1**

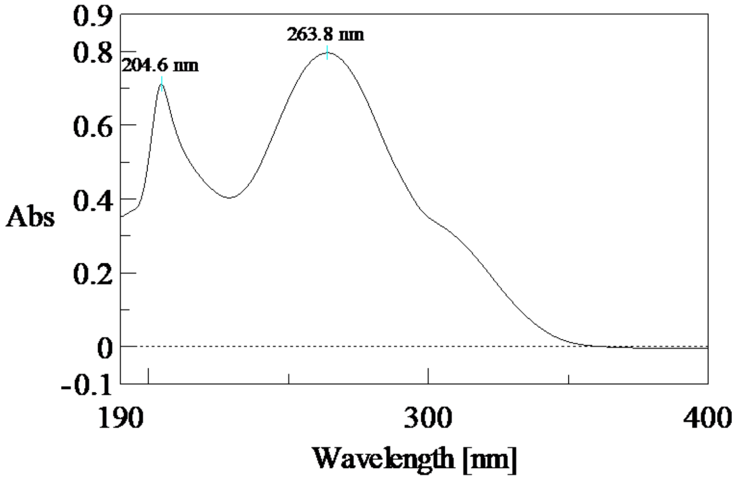

Figure S1-11 UV spectrum of compound **1**

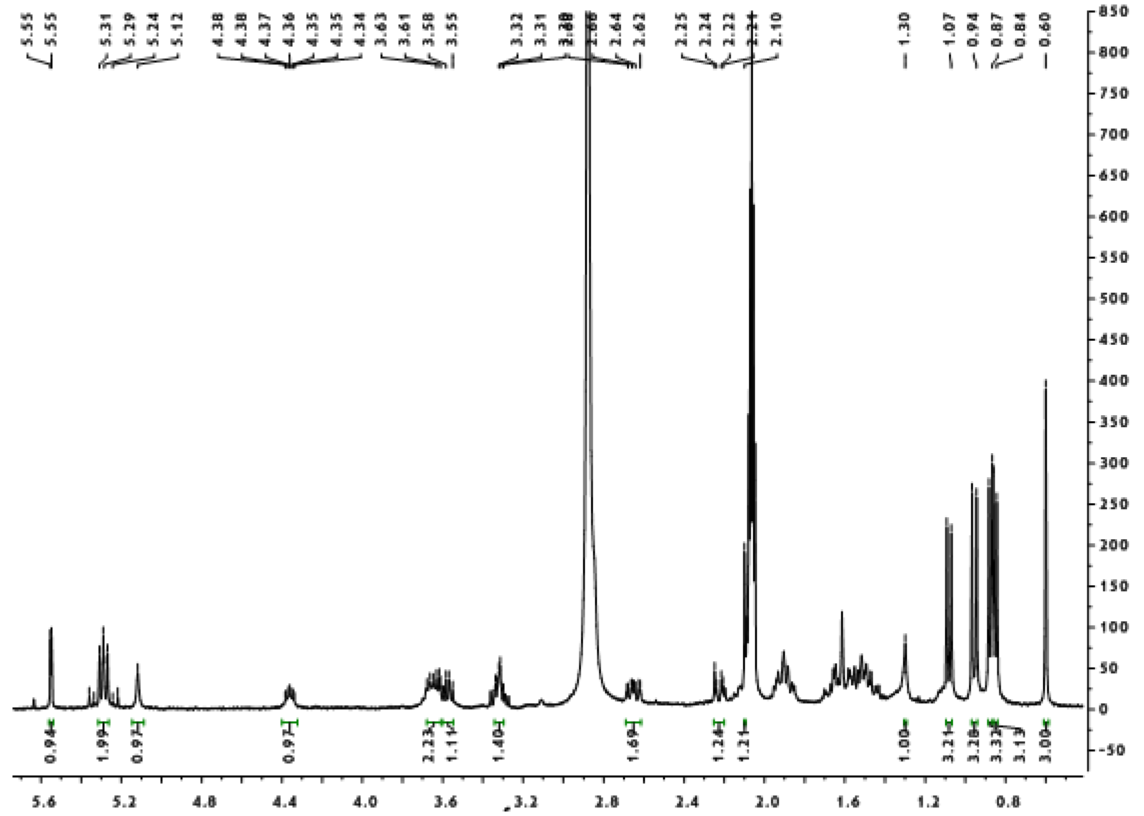

Figure S2-1 ^{1}H NMR spectrum of compound **2** (600 MHz, in CD_3COCD_3)

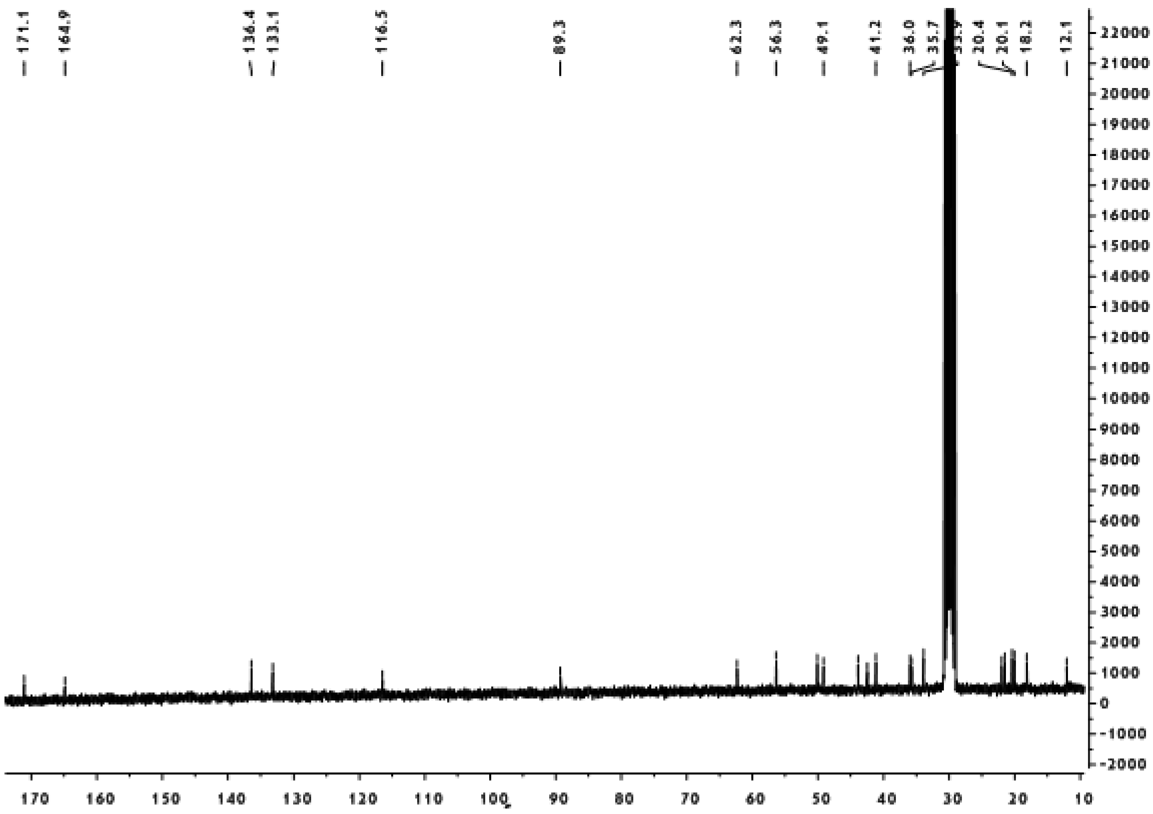

Figure S2-2 ^{13}C NMR spectrum of compound **2** (150 MHz, in CD_3COCD_3)

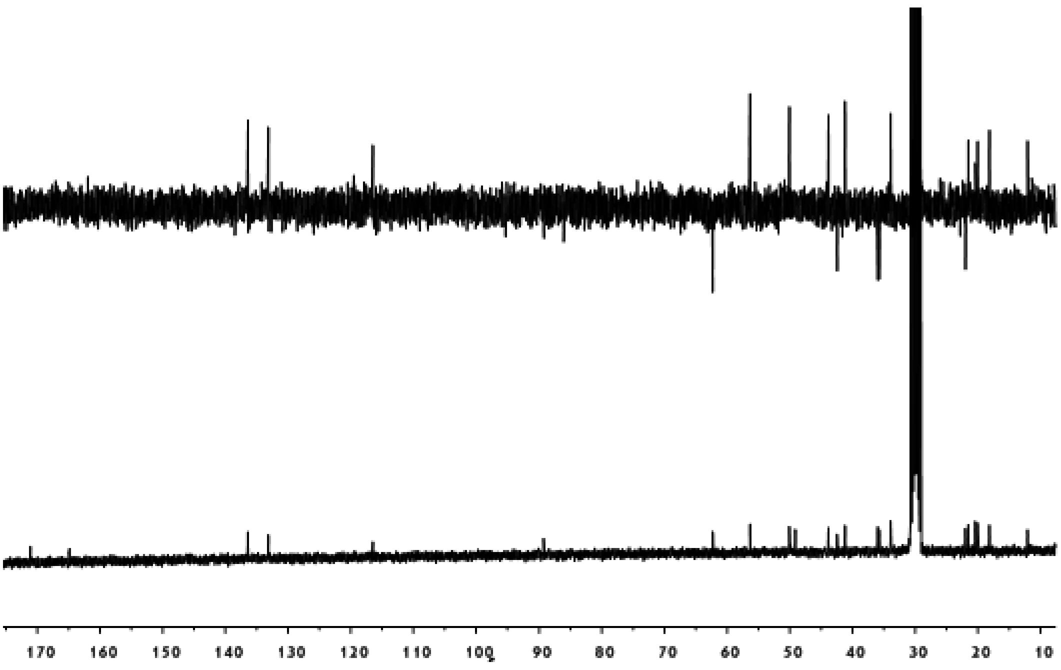

Figure S2-3 DEPT-135 spectrum of compound **2** (150 MHz, in CD_3COCD_3)

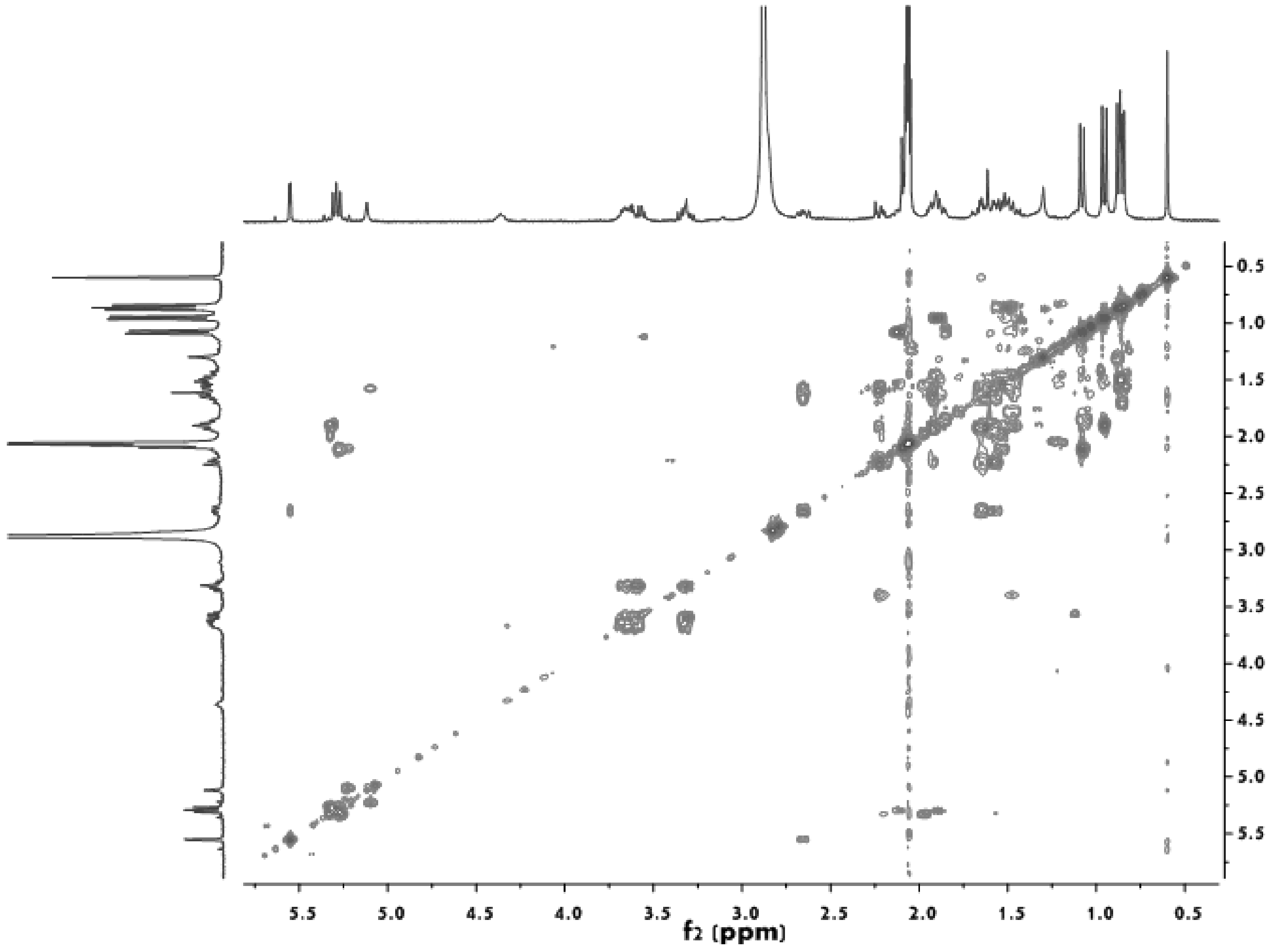

Figure S2-4 ^1H-^1H COSY spectrum of compound **2** (600 MHz, in CD_3COCD_3)

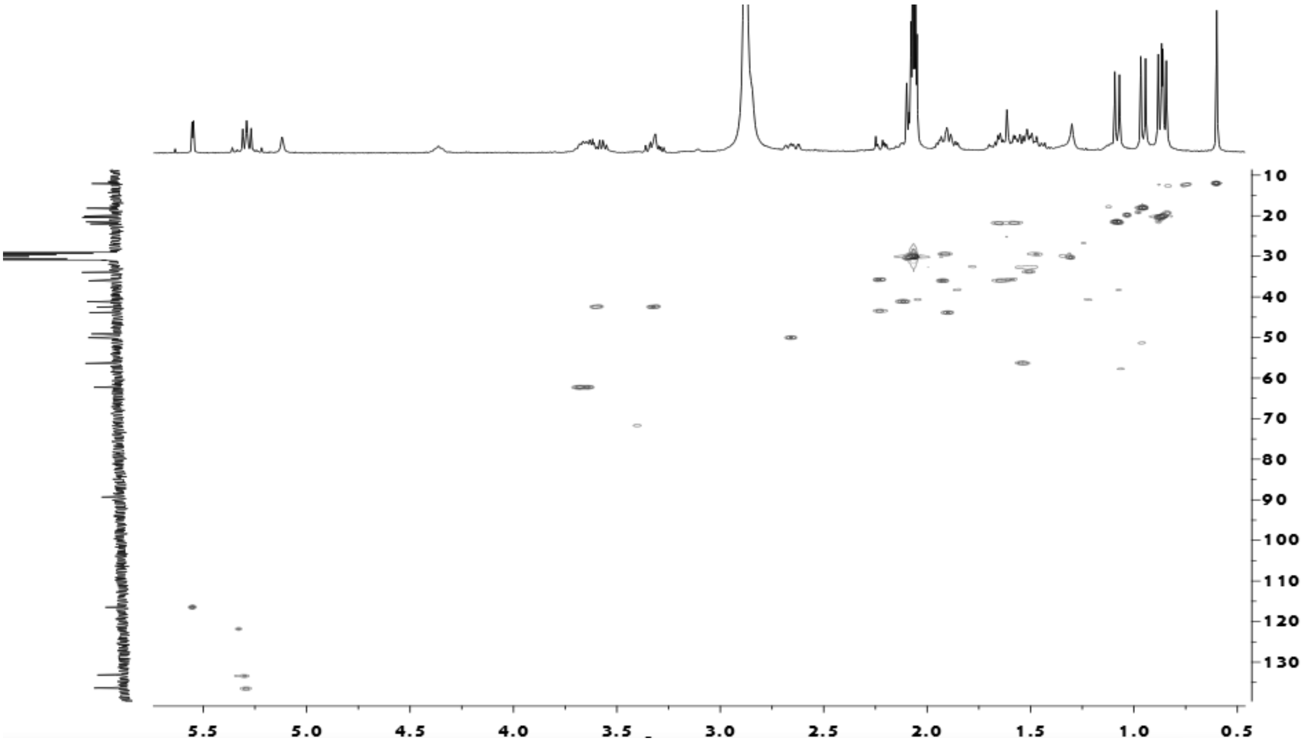

Figure S2-5 HMQC spectrum of compound **2** (600 MHz, in CD_3COCD_3)

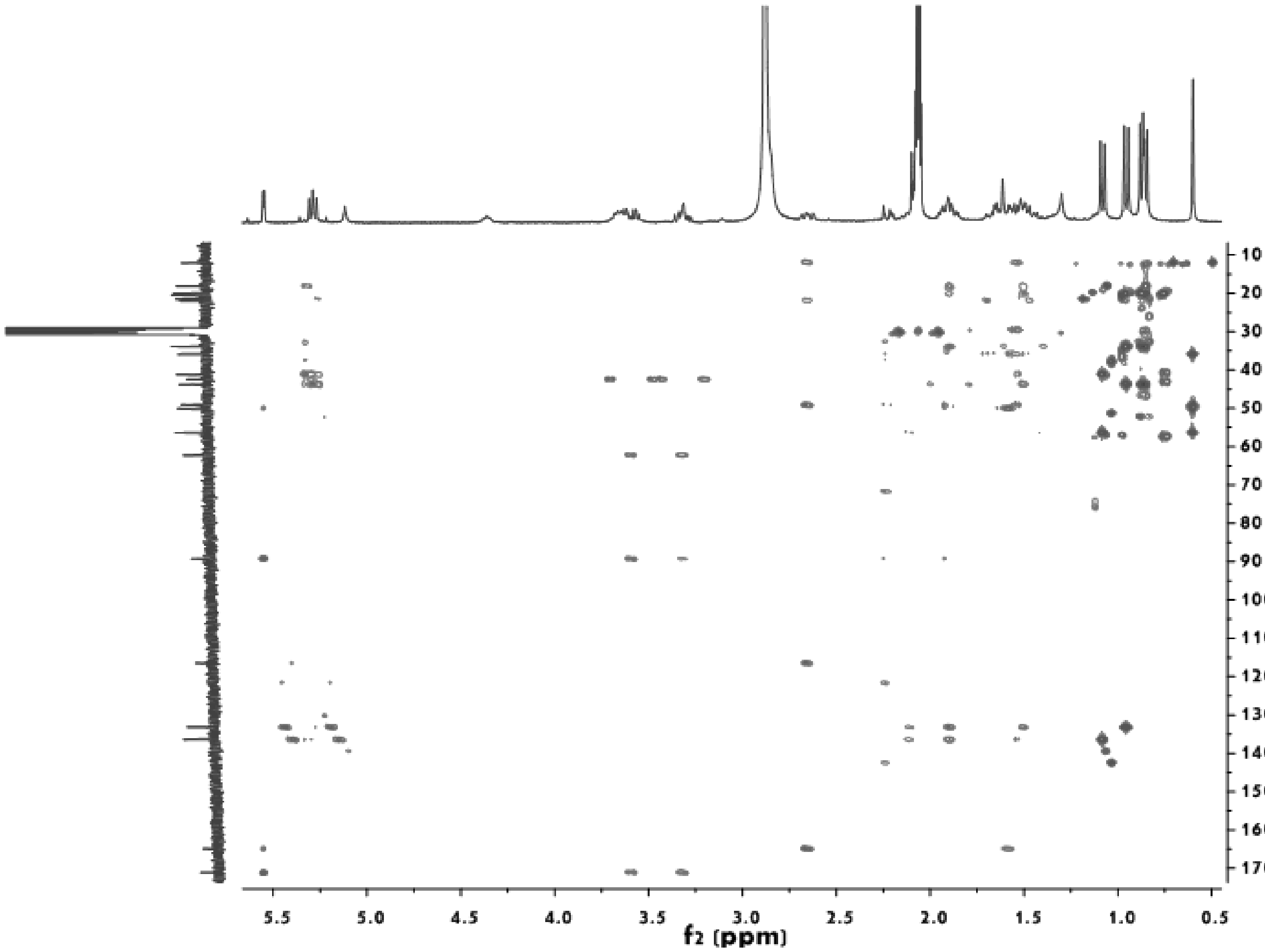

Figure 2-6 HMBC spectrum of compound **2** (600 MHz, in CD_3COCD_3)

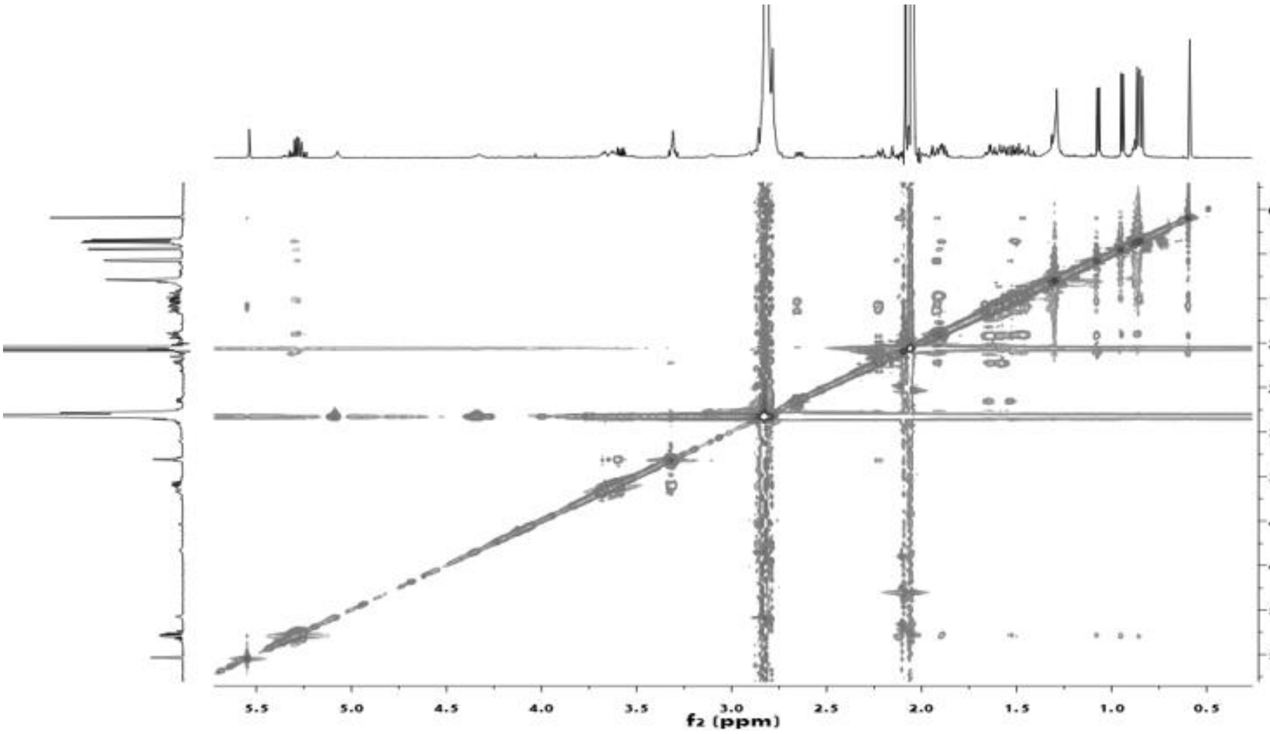

Figure S2-7-A NOESY spectrum of compound **2** (600 MHz, in CD_3COCD_3)

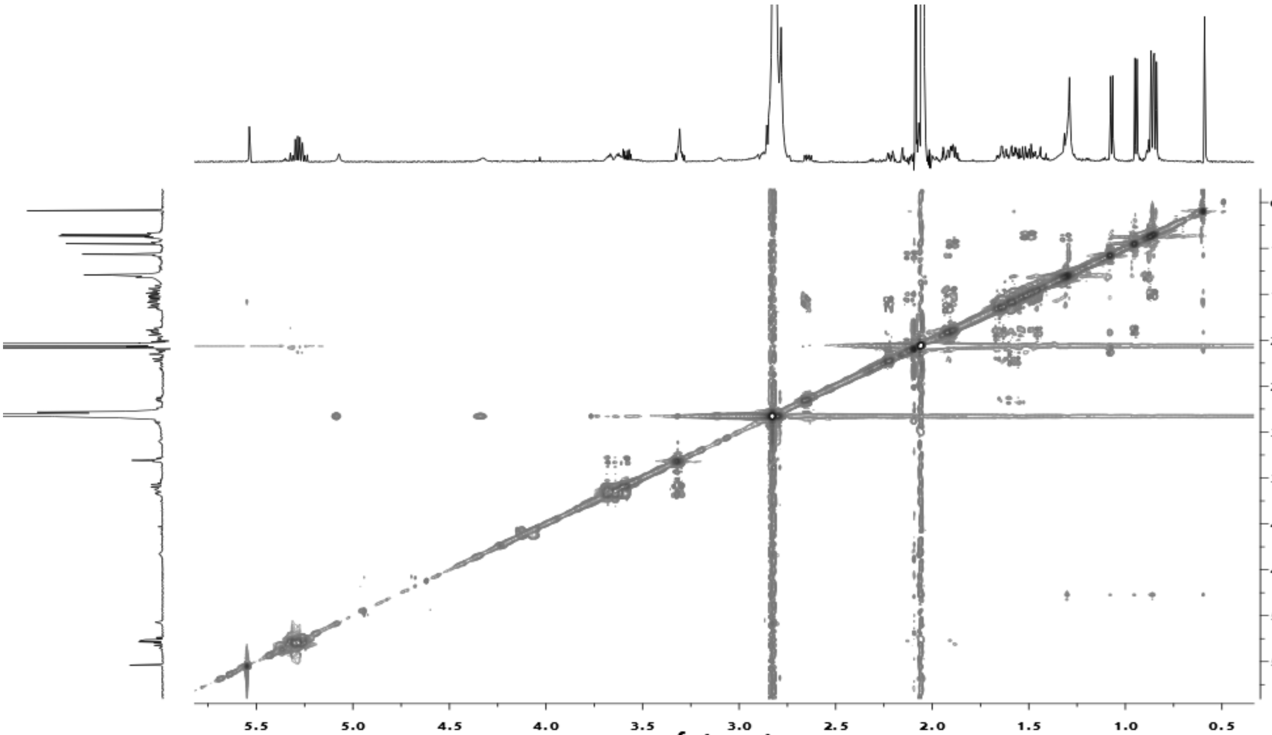

Figure S2-7-B ROESY spectrum of compound **2** (600 MHz, in CD_3COCD_3)

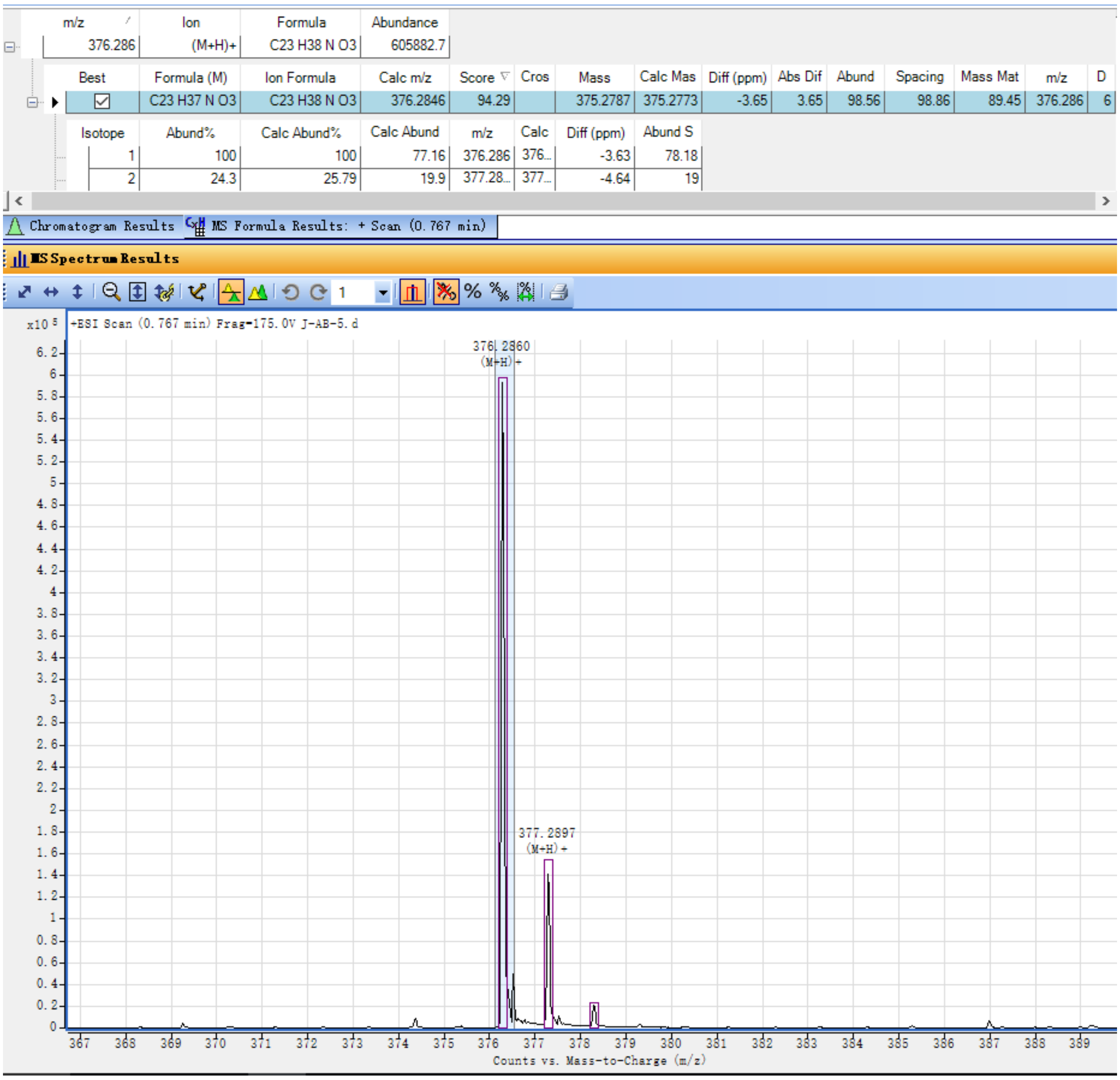

Figure S2-8 HR-ESI-MS of compound **2**

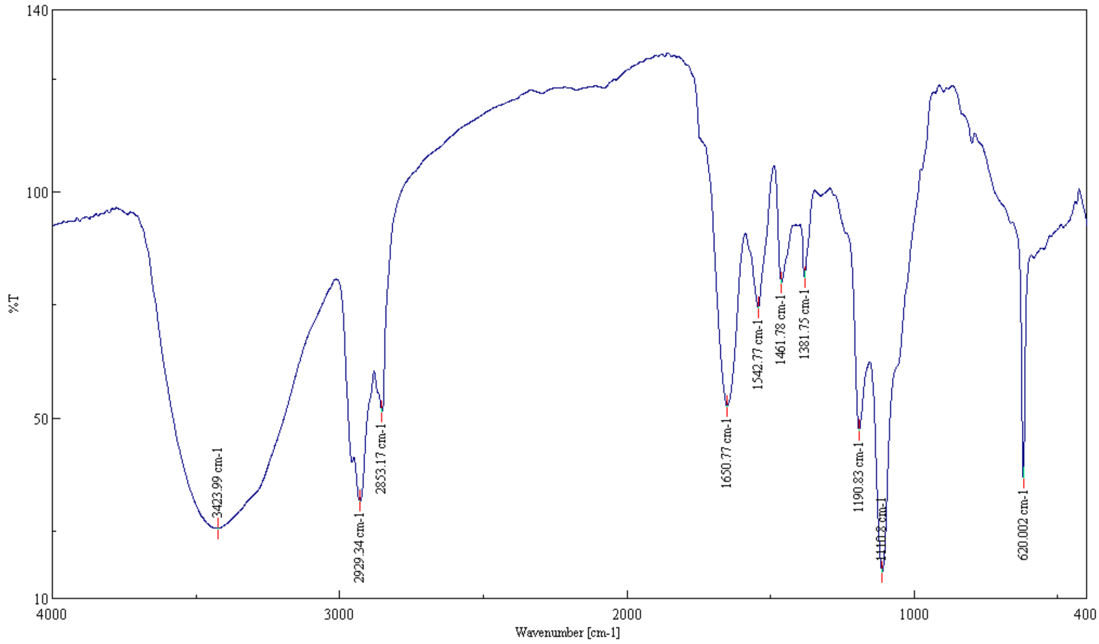

Figure S2-9 IR spectrum of compound **2**

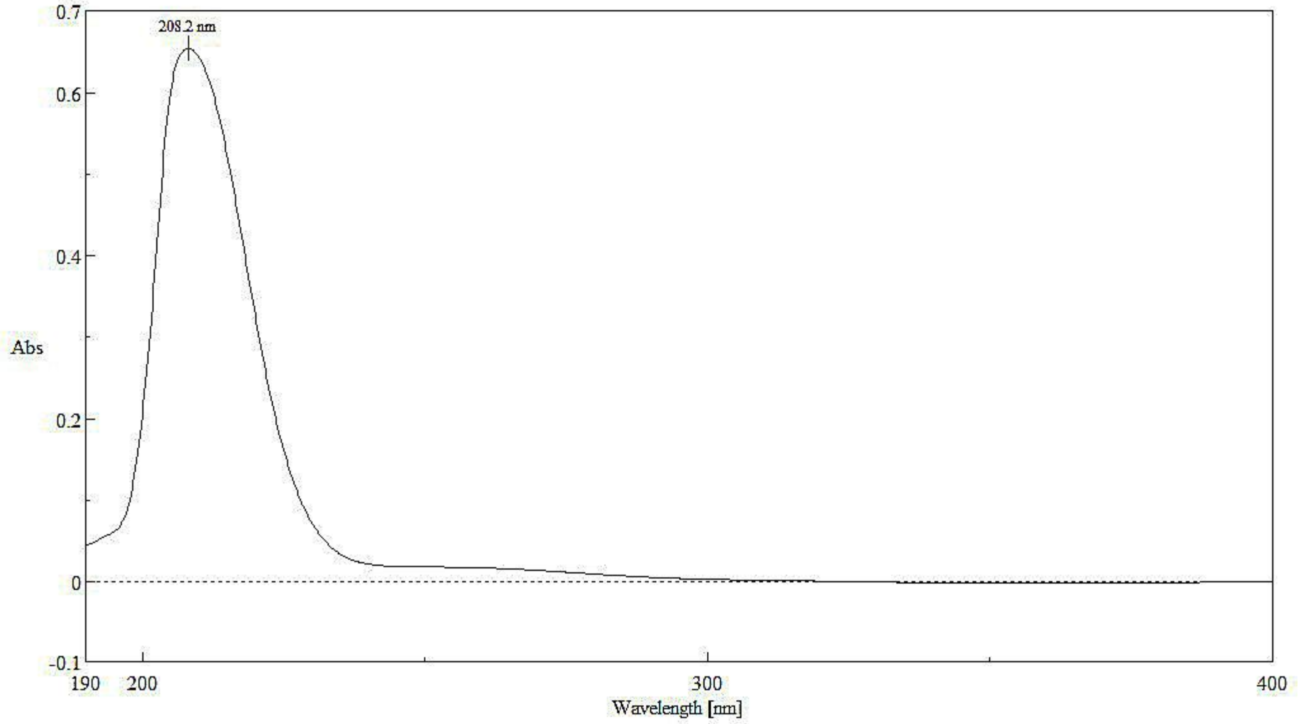

Figure S2-10 UV spectrum of compound **2**

X-ray crystallograpgic data of Compound 1

Table S1. Crystal data and structure refinement for **1.**

Identification code	zhangyangmei_h-3c
Empirical formula	$C_{23}H_{35}NO_2$
Formula weight	357.52
Temperature/K	173.00(10)
Crystal system	monoclinic
Space group	C2
a/Å	21.7844(3)
b/Å	7.23929(11)
c/Å	13.14751(20)
α/°	90.00
β/°	93.2128(13)
γ/°	90.00
Volume/Å^3	2070.15(5)
Z	4
ρ_{calc}g/cm^3	1.147
μ/mm^{-1}	0.555
F(000)	784.0

Crystal size/mm^3	0.3 × 0.28 × 0.24
Radiation	CuKα (λ = 1.54184)
2Θ range for data collection/°	8.14 to 125.64
Index ranges	-24 ≤ h ≤ 24, -8 ≤ k ≤ 8, -15 ≤ l ≤ 15
Reflections collected	16181
Independent reflections	3274 [R_{int} = 0.0282, R_{sigma} = 0.0180]
Data/restraints/parameters	3274/1/241
Goodness-of-fit on F^2	1.058
Final R indexes [I>=2σ (I)]	R_1 = 0.0286, wR_2 = 0.0747
Final R indexes [all data]	R_1 = 0.0290, wR_2 = 0.0750
Largest diff. peak/hole / e Å^{-3}	0.15/-0.12
Flack parameter	0.1(2)

Table S2. Fractional Atomic Coordinates (×10^4) and Equivalent Isotropic Displacement Parameters (Å^2×10^3) for **1**. U_{eq} is defined as 1/3 of of the trace of the orthogonalised U_{IJ} tensor.

Atom	*x*	*y*	*z*	U(eq)
C1’	1829.2(6)	-2878(2)	1272.7(11)	36.2(4)
C2	1272.9(7)	117(2)	977.9(10)	33.3(3)
C2’	1963.5(6)	-4002(3)	344.7(11)	38.5(4)
C3	642.0(7)	773(2)	1076.6(10)	33.3(3)
C4	294.0(6)	-661(2)	1331.6(10)	29.0(3)
C5	678.5(6)	-2309(2)	1403.6(10)	30.3(3)
C6	469.2(6)	-3966(2)	1650.8(11)	34.6(3)
C7	-192.7(6)	-4244(2)	1913.6(11)	33.2(3)
C8	-488.1(6)	-2444(2)	2273.2(10)	27.7(3)
C9	-372.5(6)	-931(2)	1477.7(10)	28.9(3)
C10	-770.1(7)	696(2)	1767.7(12)	35.5(3)
C11	-1334.9(7)	-252(2)	2198.2(12)	35.6(3)
C12	-1200.8(6)	-2352(2)	2233(1)	28.1(3)
C13	-216.6(6)	-1938(2)	3339.6(10)	35.8(3)
C14	-1553.0(6)	-3359(2)	3049.8(11)	30.5(3)
C15	-1483.4(7)	-5454(2)	2982.7(13)	41.3(4)
C16	-2226.2(6)	-2869(2)	2945.2(10)	29.4(3)
C17	-2542.7(6)	-2171(2)	3676.1(10)	29.5(3)
C18	-3214.5(6)	-1704(2)	3618(1)	29.0(3)
C19	-3579.3(7)	-3010(2)	4286.9(11)	33.7(3)
C20	-4257.5(8)	-2498(3)	4292.2(17)	60.0(5)
C21	-3521.0(7)	-5012(2)	3956.0(12)	39.9(4)

C22	-3292.8(8)	315(2)	3930.9(12)	41.1(4)
N1	1273.6(5)	-1751.3(19)	1162.4(9)	34.0(3)
O1	1733.3(5)	1023.3(17)	778.0(8)	42.2(3)
O2	1496.1(5)	-5300.3(18)	77.5(10)	49.9(3)

Table S3. Anisotropic Displacement Parameters ($Å^2 \times 10^3$) for **1**. The Anisotropic displacement factor exponent takes the form: $-2\pi^2[h^2a^{*2}U_{11}+2hka^*b^*U_{12}+\cdots]$.

Atom	U_{11}	U_{22}	U_{33}	U_{23}	U_{13}	U_{12}
C1'	24.1(7)	47.9(10)	36.8(8)	5.3(7)	4.2(6)	0.4(6)
C2	30.7(7)	39.5(9)	30.2(7)	-1.1(6)	7.8(6)	-7.6(6)
C2'	26.4(7)	48.4(10)	41.2(8)	5.5(7)	6.9(6)	4.6(7)
C3	33.1(7)	32.6(8)	34.9(7)	-0.7(6)	8.8(6)	-2.9(7)
C4	28.9(7)	30.6(8)	28.0(7)	-2.2(6)	6.2(5)	-2.4(6)
C5	25.2(7)	34.9(8)	31.3(7)	-1.2(6)	6.4(5)	-1.5(6)
C6	26.9(7)	31.9(8)	45.8(8)	-0.6(7)	9.7(6)	2.1(6)
C7	27.1(7)	28.7(8)	44.5(8)	1.0(6)	8.3(6)	-0.5(6)
C8	23.0(7)	29.8(8)	30.7(7)	-0.9(6)	5.1(5)	-2.1(6)
C9	26.3(7)	29.6(7)	31.4(7)	0.7(6)	5.9(5)	-0.8(6)
C10	32.4(7)	30.8(8)	44.5(8)	3.8(6)	12.0(6)	2.5(7)
C11	28.8(7)	33.1(8)	45.9(8)	3.9(7)	10.4(6)	1.6(6)
C12	23.8(7)	31.3(7)	29.7(6)	-1.3(6)	4.9(5)	-0.1(6)
C13	28.0(7)	45.1(10)	34.5(7)	0.2(7)	3.1(6)	-5.9(6)
C14	26.0(7)	35.7(8)	30.4(7)	0.0(6)	6.8(5)	-3.0(6)
C15	36.7(8)	36.8(10)	52.1(9)	6.4(7)	17.3(7)	-0.9(7)
C16	27.6(7)	30.8(8)	30.2(7)	-1.3(6)	5.3(5)	-5.7(6)
C17	28.4(7)	31.3(8)	29.2(6)	-0.4(6)	3.5(5)	-4.4(6)
C18	28.8(7)	29.8(7)	28.7(7)	-2.5(6)	4.8(5)	1.8(6)
C19	30.2(8)	37.4(9)	34.3(7)	-0.7(6)	8.4(6)	-3.4(6)
C20	36.9(9)	58.4(13)	87.4(14)	-1.4(11)	26.9(9)	2.3(9)
C21	36.9(8)	35.1(9)	48.0(9)	2.5(7)	3.7(7)	-5.7(7)
C22	48.8(9)	31.9(9)	43.2(9)	-2.6(7)	7.7(7)	2.9(7)
N1	23.5(6)	39.7(8)	39.4(6)	-0.9(5)	8.5(5)	-3.4(5)
O1	31.2(5)	48.1(7)	48.5(6)	2.1(5)	10.5(4)	-12.3(5)
O2	37.8(6)	50.0(7)	61.6(7)	-7.5(6)	-0.1(5)	1.6(5)

Table S4. Bond Lengths for 1.

Atom	Atom	Length/Å	Atom	Atom	Length/Å

C1’	C2’	1.509(2)	C8	C12	1.5520(18)
C1’	N1	1.4598(19)	C8	C13	1.5350(18)
C2	C3	1.467(2)	C9	C10	1.523(2)
C2	N1	1.374(2)	C10	C11	1.544(2)
C2	O1	1.2392(17)	C11	C12	1.548(2)
C2’	O2	1.415(2)	C12	C14	1.5380(19)
C3	C4	1.339(2)	C14	C15	1.528(2)
C4	C5	1.458(2)	C14	C16	1.5074(19)
C4	C9	1.4880(18)	C16	C17	1.315(2)
C5	C6	1.330(2)	C17	C18	1.4999(19)
C5	N1	1.4109(18)	C18	C19	1.5420(19)
C6	C7	1.5149(18)	C18	C22	1.531(2)
C7	C8	1.540(2)	C19	C20	1.524(2)
C8	C9	1.5446(19)	C19	C21	1.520(2)

Table S5. Bond Angles for 1.

Atom	Atom	Atom	Angle/°	Atom	Atom	Atom	Angle/°
N1	C1’	C2’	114.73(12)	C4	C9	C10	120.22(13)
N1	C2	C3	107.15(12)	C10	C9	C8	105.19(11)
O1	C2	C3	128.39(15)	C9	C10	C11	102.97(12)
O1	C2	N1	124.46(14)	C10	C11	C12	107.10(12)
O2	C2’	C1’	112.92(12)	C11	C12	C8	103.30(11)
C4	C3	C2	108.53(14)	C14	C12	C8	119.54(11)
C3	C4	C5	108.62(12)	C14	C12	C11	112.66(11)
C3	C4	C9	134.80(15)	C15	C14	C12	112.05(12)
C5	C4	C9	116.43(13)	C16	C14	C12	110.23(12)
C6	C5	C4	123.39(13)	C16	C14	C15	109.10(12)
C6	C5	N1	130.16(14)	C17	C16	C14	124.82(13)
N1	C5	C4	106.45(13)	C16	C17	C18	126.84(13)
C5	C6	C7	121.29(13)	C17	C18	C19	111.42(12)
C6	C7	C8	112.15(12)	C17	C18	C22	108.95(13)
C7	C8	C9	107.78(11)	C22	C18	C19	111.32(12)
C7	C8	C12	117.41(11)	C20	C19	C18	112.58(13)
C9	C8	C12	98.47(10)	C21	C19	C18	111.55(12)
C13	C8	C7	109.64(12)	C21	C19	C20	109.27(14)
C13	C8	C9	112.36(12)	C2	N1	C1’	124.07(13)
C13	C8	C12	110.75(11)	C2	N1	C5	109.22(12)
C4	C9	C8	112.21(11)	C5	N1	C1’	125.80(13)

Table 6 Hydrogen Bonds for zhangyangmei_h-3c.

D	H	A	d(D-H)/Å	d(H-A)/Å	d(D-A)/Å	D-H-A/°
O2	H2	O1[1]	0.82	2.04	2.8540(18)	171.9

[1]+X, -1+Y, +Z

Table S7. Torsion Angles for **1**.

A	B	C	D	Angle/˚	A	B	C	D	Angle/˚
C2	C3	C4	C5	-0.39(16)	C9	C4	C5	N1	175.47(11)
C2	C3	C4	C9	-175.50(14)	C9	C8	C12	C11	-42.28(13)
C2'	C1'	N1	C2	-101.68(17)	C9	C8	C12	C14	-168.36(12)
C2'	C1'	N1	C5	90.38(17)	C9	C10	C11	C12	5.61(16)
C3	C2	N1	C1'	-171.39(12)	C10	C11	C12	C8	23.58(15)
C3	C2	N1	C5	-1.73(15)	C10	C11	C12	C14	153.95(12)
C3	C4	C5	C6	179.55(14)	C11	C12	C14	C15	172.46(13)
C3	C4	C5	N1	-0.66(15)	C11	C12	C14	C16	50.77(16)
C3	C4	C9	C8	-149.98(16)	C12	C8	C9	C4	179.86(12)
C3	C4	C9	C10	-25.5(2)	C12	C8	C9	C10	47.47(13)
C4	C5	C6	C7	-2.1(2)	C12	C14	C16	C17	-124.22(16)
C4	C5	N1	C1'	170.94(13)	C13	C8	C9	C4	63.21(15)
C4	C5	N1	C2	1.50(15)	C13	C8	C9	C10	-69.18(14)
C4	C9	C10	C11	-161.03(12)	C13	C8	C12	C11	75.61(14)
C5	C4	C9	C8	35.20(16)	C13	C8	C12	C14	-50.47(17)
C5	C4	C9	C10	159.62(13)	C14	C16	C17	C18	-178.44(14)
C5	C6	C7	C8	-22.87(19)	C15	C14	C16	C17	112.35(17)
C6	C5	N1	C1'	-9.3(2)	C16	C17	C18	C19	111.26(17)
C6	C5	N1	C2	-178.72(15)	C16	C17	C18	C22	-125.53(16)
C6	C7	C8	C9	50.82(15)	C17	C18	C19	C20	176.62(14)
C6	C7	C8	C12	160.73(12)	C17	C18	C19	C21	-60.11(16)
C6	C7	C8	C13	-71.76(15)	C22	C18	C19	C20	54.77(18)
C7	C8	C9	C4	-57.69(14)	C22	C18	C19	C21	178.04(14)
C7	C8	C9	C10	169.92(11)	N1	C1'	C2'	O2	-61.62(18)
C7	C8	C12	C11	-157.42(12)	N1	C2	C3	C4	1.32(16)
C7	C8	C12	C14	76.50(16)	N1	C5	C6	C7	178.20(14)

C8	C9	C10	C11	-33.34(15)	O1	C2	C3	C4	-178.00(14)
C8	C12	C14	C15	-66.00(17)	O1	C2	N1	C1'	8.0(2)
C8	C12	C14	C16	172.31(12)	O1	C2	N1	C5	177.62(13)
C9	C4	C5	C6	-4.3(2)					

Table S8. Hydrogen Atom Coordinates (Å×10^4) and Isotropic Displacement Parameters (Å^2×10^3) for **1**.

Atom	*x*	*y*	*z*	U(eq)
H1'A	1792	-3711	1844	43
H1'B	2177	-2073	1436	43
H2'A	2011	-3174	-225	46
H2'B	2349	-4653	474	46
H3	506	1981	979	40
H6	734	-4973	1663	41
H7A	-208	-5166	2447	40
H7B	-428	-4706	1319	40
H9	-552	-1393	827	35
H10A	-888	1445	1177	43
H10B	-557	1465	2278	43
H11A	-1700	-3	1764	43
H11B	-1401	208	2877	43
H12	-1348	-2859	1572	34
H13A	219	-1751	3315	54
H13B	-293	-2923	3805	54
H13C	-406	-825	3566	54
H14	-1388	-2954	3722	37
H15A	-1767	-6037	3414	62
H15B	-1071	-5796	3200	62
H15C	-1568	-5845	2291	62
H16	-2433	-3077	2317	35
H17	-2327	-1943	4294	35
H18	-3372	-1842	2909	35
H19	-3404	-2910	4988	40
H20A	-4298	-1306	4601	90
H20B	-4470	-3402	4673	90
H20C	-4431	-2461	3605	90
H21A	-3699	-5154	3276	60
H21B	-3733	-5794	4410	60
H21C	-3095	-5351	3973	60
H22A	-3205	436	4652	62

H22B	-3708	701	3762	62
H22C	-3014	1075	3575	62
H2	1592	-6312	317	75
